# The global, regional, and national burden of acute pancreatitis in 204 countries and territories, 1990–2019

**DOI:** 10.1186/s12876-021-01906-2

**Published:** 2021-08-25

**Authors:** Chang-li Li, Meng Jiang, Chun-qiu Pan, Jian Li, Li-gang Xu

**Affiliations:** 1 Department of Geratology, Hubei Provincial Hospital of Integrated Chinese and Western Medicine, Wuhan, 430015 China; 2 Department of Medical Ultrasound, Tongji Hospital, Tongji Medical College, Huazhong University of Science and Technology, Wuhan, 430030 China; 3 Department of Emergency Medicine, Nanfang Hospital, Southern Medical University, Guangzhou, 510515 China; 4 Department of Critical Care Medicine, Wuhan Central Hospital, Tongji Medical College, Huazhong University of Science and Technology, Wuhan, 430000 China

**Keywords:** Acute pancreatitis, Epidemiology, Disease prevention, Global burden of disease study 2019

## Abstract

**Background:**

Acute pancreatitis is a common and potentially lethal gastrointestinal disease, but literatures for the disease burden are scarce for many countries. Understanding the current burden of acute pancreatitis and the different trends across various countries is essential for formulating effective preventive intervenes. We aimed to report the incidence, mortality, and disability-adjusted life-years (DALYs) caused by acute pancreatitis in 204 countries and territories between 1990 and 2019.

**Methods:**

Estimates from the Global Burden of Disease Study 2019 (GBD 2019) were used to analyze the epidemiology of acute pancreatitis at the global, regional, and national levels. We also reported the correlation between development status and acute pancreatitis’ age-standardized DALY rates, and calculated DALYs attributable to alcohol etiology that had evidence of causation with acute pancreatitis. All of the estimates were shown as counts and age-standardized rates per 100,000 person-years.

**Results:**

There were 2,814,972.3 (95% UI 2,414,361.3–3,293,591.8) incident cases of acute pancreatitis occurred in 2019 globally; 1,273,955.2 (1,098,304.6–1,478,594.1) in women and 1,541,017.1 (1,307,264.4–1,814,454.3) in men. The global age-standardized incidence rate declined from 37.9/100,000 to 34.8/100,000 during 1990–2019, an annual decrease of 8.4% (5.9–10.4%). In 2019, there were 115,053.2 (104,304.4–128,173.4) deaths and 3,641,105.7 (3,282,952.5–4,026,948.1) DALYs due to acute pancreatitis. The global age-standardized mortality rate decreased by 17.2% (6.6–27.1%) annually from 1.7/100,000 in 1990 to 1.4/100,000 in 2019; over the same period, the age-standardized DALY rate declined by 17.6% (7.8–27.0%) annually. There were substantial differences in the incidence, mortality and DALYs across regions. Alcohol etiology attributed to a sizable fraction of acute pancreatitis-related deaths, especially in the high and high-middle SDI regions.

**Conclusion:**

Substantial variation existed in the burden of acute pancreatitis worldwide, and the overall burden remains high with aging population. Geographically targeted considerations are needed to tailor future intervenes to relieve the burden of acute pancreatitis in specific countries, especially for Eastern Europe.

**Supplementary Information:**

The online version contains supplementary material available at 10.1186/s12876-021-01906-2.

## Background

Acute pancreatitis is a common and potentially lethal gastrointestinal disease. Previous studies have shown a variable incidence rate, ranging from 15.0 per 100,000 in Denmark [1] to 83.7 per 100,000 in Sweden [2]. In the USA, it accounts for about 275,000 hospital admissions and $2.5 billion health care costs each year [3]. Approximately 20% of patients develop moderate to severe acute pancreatitis, which lead to a mortality rate of 20–40% [4–6].

The global incidence of acute pancreatitis cited in previous publications was presented as a wide range of estimates, mainly because they were based on heterogeneous study populations and varying methodological quality. In a meta-analysis, Xiao et al [7] pooled the data from general population-based cohort studies, reported that the incidence and mortality rates of acute pancreatitis were 33.7 cases and 1.6 deaths per 100,000-person years. However, the original studies included in this systematic review were confined to only five countries or territories (Sweden, Denmark, Taiwan, USA and United Kingdom), all of which were high-income regions and may not be representative of the global population. In addition, the reported incidence and mortality rates were not age-standardized, which might skew the comparisons between different regions.

Global efforts using appropriate preventive and treatment approaches to reduce the morbidity and mortality of acute pancreatitis require timely information about the burden and their risk factors. However, the current analyses on the epidemiology of acute pancreatitis were based exclusively on limited local data [1, 8–15], which inevitably subjected to selection bias and could not describe the disease burden around the globe in a robust manner. The Global Burden of Disease study (GBD), with its broad collection of data sources and the state-of-the-art statistical modelling approaches [16–18] provides us a unique opportunity to deliver the most comprehensive estimates of acute pancreatitis’ burden to date. Since GBD 2017 [19], no comprehensive update of epidemiological levels and trends on acute pancreatitis have been released. In prior GBD analysis conducted by Ouyang et al. [20], the acute and chronic pancreatitis were modelled together; but in this study, we aimed to analyze acute pancreatitis separately based on GBD 2019. In this study, we summarized GBD 2019 findings on acute pancreatitis’ epidemiology in 204 countries and territories, presented the temporal and geographical trends in terms of incidence, mortality, disability-adjusted life-years (DALYs), and their age-standardized rates by sex and location during 1990–2019.

## Methods

### Data acquisition

Acute pancreatitis in GBD 2019 were identified according to the ICD-10 code K85 and ICD-9 code 577.0. For this study, we used GBD 2019 vital registration (19,618 site-years of data) and verbal autopsy (374 site-years) data sources that provided a representative partial or complete sample of incidence or mortality. Information about the data sources used for each location in this study can be found on the GBD 2019 Data Input Sources Tool website (http://ghdx.healthdata.org/gbd-2019/data-input-sources). The GBD 2019 database contains the statistical data of 369 diseases and 87 risk factors in 204 countries and territories [21, 22]. The data of acute pancreatitis in GBD 2019 including incidence, mortality, DALYs, and corresponding age-standardized rates by sex and location for each year from 1990 through 2019 were obtained publicly from the Global Health Data Exchange (GHDx) website (http://ghdx.healthdata.org/gbd-results-tool). More details on the case definition, input data, data processing, and modeling strategy, as well as the differences between GBD 2019 and GBD 2017 on acute pancreatitis are provided in Additional file 1.

### Socio-demographic index (SDI)

The SDI for all the 204 countries and territories during 1990–2019 was downloaded from the GHDx website for the following correlation analysis. SDI is a compound indicator of development status, created according to a country’s total fertility rate for females younger than 25 years, mean education for those aged 15 years and older, and lag-distributed income per capita. Countries and territories were ranked into high, high-middle, middle, low-middle, and low SDI categories. The methods for SDI generation are detailed in previous GBD publications [23, 24].

### Statistical analysis

The standardized methods of the GBD 2019 have been extensively reported [22]. Three main standardized tools—Cause of Death Ensemble model (CODEm), DisMod-MR 2.1 and spatiotemporal Gaussian process regression (ST-GPR)—were used to generate estimates for each quantity of interest by age, sex, location, and year. Briefly, CODEm was used to analyze death-related data. DisMod-MR 2.1 based on Bayesian meta-regression was applied to evaluate all available data on incidence and DALY. The expected relationship between SDI and age-standardized incidence, mortality and DALY rates were determined by fitting a ST-GPR for all locations from 1990 to 2019 [22]. The 95% uncertainty intervals (UIs) were determined for each parameter using the 25th and 975th of the 1000 ordered draws based on the posterior distribution.

The annual absolute number of incident cases, deaths, DALYs, and corresponding age-standardized rates were applied to delineate the burden of acute pancreatitis at the global, regional, and national levels. The age-standardized rates could exclude the impact from imbalance in population quantity and age distribution. DALYs was the summation of the years lived with disability (YLDs) and the years of life lost (YLLs). Moreover, the corresponding estimated annual percentage change (EAPC) values of age-standardized incidence/mortality rate (ASIR/ASMR) and age-standardized DALY rate per 100,000 people were employed to reflect the spatiotemporal trends of acute pancreatitis’ burden. In the formula *Y* = *α* + *βX*, *Y* refers to lg (age-standardized rate) while *X* means the calendar year. Then EAPC values were calculated by the formula *EAPC* = 100* (10^β^—1). In the case that both EAPC value and its 95% UI above zero, the corresponding age-standardized rate was in an upward trend and vice versa. Lastly, we searched the GBD 2019 database for potential risk factors that contributing to acute pancreatitis-related fatality. This study is compliant with the Guidelines for Accurate and Transparent Health Estimates Reporting (GATHER) [25].

## Results

### Incidence of acute pancreatitis

**Fig. 1:**
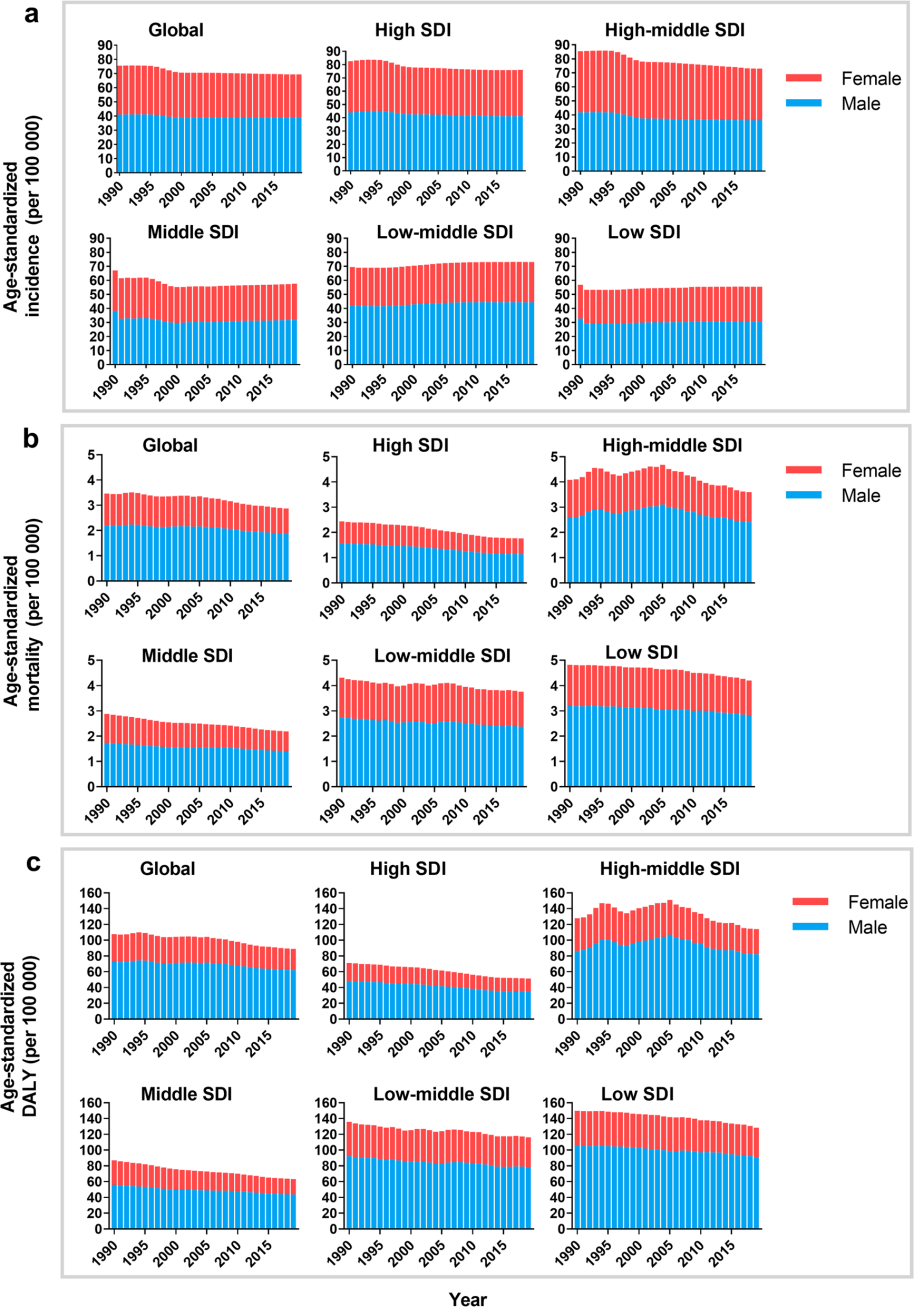
The change trends of age-standardized acute pancreatitis’ incidence (**a**), mortality (**b**), and DALY rates (**c**) per 100,000 person-years from 1990 to 2019. DALY: disability-adjusted life-year; SDI: social demographic index

There were 1,727,789.3 (95% UI 1,452,132.4–2,059,695.3) acute pancreatitis occurred in 1990 and 2,814,972.3 (95% UI 2,414,361.3–3,293,591.8) occurred in 2019 in the globe, with an increase of 62.9% (Table 1**)**. However, the ASIR declined by an average of 8.4% (95% UI 5.9–10.4%) annually during the same period, decreased from 37.9 to 34.8 per 100,000. Males were more likely to suffer from acute pancreatitis than females (41.0 vs 34.5 per 100,000 in 1990, and 38.8 vs 30.6 per 100,000 in 2019) (Table 1, Fig. 1a).

**Table 1:** The incidence of acute pancreatitis in 1990/2019 and temporal trends

Location	Incident cases (95% UI)		Age-standardized incidence rate (per 100,000)		EAPC of incidence rate
	1990	2019	1990	2019	1990–2019 (%)
Global	1,727,789.3 (1,452,132.4–2,059,695.3)	2,814,972.3 (2,414,361.3–3,293,591.8)	37.9 (32–44.6)	34.8 (29.8–40.7)	– 8.4 (– 10.4 to – 5.9)
Sex					
Male	929,028.7 (774,587.6–1,115,575.6)	1,541,017.1 (1,307,264.4–1,814,454.3)	41.0 (34.3–48.5)	38.8 (33.1–45.5)	– 5.4 (– 7.5 to – 2.8)
Female	798,760.6 (677,669.1–943,983.6)	1,273,955.2 (1,098,304.6–1,478,594.1)	34.5 (29.2–40.3)	30.6 (26.4–35.6)	– 11.2 (– 13.2 to – 8.8)
SDI factor					
High SDI	390,419.1 (335,676.7–452,156.8)	533,633.2 (477,729.8–597,075.3)	41.3 (35.4–48.0)	38.1 (33.9–42.7)	– 7.9 (– 11.8 to – 3.1)
High-middle SDI	482,857.1 (406,521.4–568,658.8)	667,222.6 (569,618.5–769,408.6)	43.2 (36.6–50.6)	36.7 (31.5–42.6)	– 15.0 (– 16.6 to – 13.1)
Middle SDI	455,054.8 (373,090.6–554,469.3)	735,082.3 (616,164.7–867,454.5)	33.7 (27.8–40.1)	28.8 (24.4–34.2)	– 14.3 (– 16.7 to – 11.4)
Low-middle SDI	297,240.1 (244,736.5–361,676.3)	598,531.4 (497,030.5–720,521.9)	34.9 (29.0–41.8)	36.5 (30.7–43.7)	4.6 (3.0–6.4)
Low SDI	101,572 (83,136.7–123,390.1)	224,224.5 (183,906.9–270,951.5)	28.4 (23.8–33.7)	27.7 (23.4–32.9)	– 2.4 (– 3.5 to – 1.3)
Region					
Andean Latin America	12,751.6 (10,888.9–14,964.7)	26,446.1 (23,242.3–30,101.7)	45.8 (39.6–52.9)	43.5 (38.4–49.3)	– 5.0 (– 7.3 to – 2.6)
Australasia	8618.4(7250.0–10,167.5)	14,394.8 (12,298.7–16,770.4)	38.6 (32.5–45.3)	37.6 (31.7–44)	– 2.8 (– 5.2 to – 0.3)
Caribbean	8807.7 (7294.7–10,668.9)	14,155.4 (11,772.9–16,692.7)	28.8 (23.9–34.3)	28.4 (23.5–33.6)	– 1.7 (– 2.9 to – 0.4)
Central Asia	18,365.6 (15,467.5–21,700.0)	29,035.3 (24,076.9–34,326.1)	33.7 (28.4–39.4)	32.9 (27.7–38.5)	– 2.4 (– 3.4 to – 1.1)
Central Europe	67,402.6 (57,280.6–78,292.3)	73,015.8 (64,542.3–82,156.6)	49.4 (42–57.3)	45.2 (40.1–50.9)	– 8.6 (– 12.5 to – 3.2)
Central Latin America	46,647.8 (39,437.0–55,591.9)	96,777.3 (83,302.4–112,767.0)	37.6 (32.1–44)	38.6 (33.3–44.7)	2.6 (1.0–4.4)
Central Sub-Saharan Africa	7427.2 (6052.5–9165.5)	18,256.3 (14,929.3–22,374.1)	21.1 (17.6–25)	20.8 (17.5–24.7)	– 1.0 (– 2.9 to – 0.9)
East Asia	396,687.7 (322,719.1–482,300.5)	526,066.5 (444,786.6–615,193.3)	38 (31.1–45.4)	27.6 (23.5–32.2)	– 27.4 (– 30.4 to – 23.8)
Eastern Europe	182,577.6 (155,056.7–211,692.2)	221,945.2 (188,142.5–258,013.8)	71.2 (60.8–82.9)	79.6 (68.2–92.5)	11.7 (10.8–12.7)
Eastern Sub-Saharan Africa	25,232.9 (20,531.3–31,140.5)	57,965.7 (46,838.7–71,275.5)	21.1 (17.7–25.2)	21.1 (17.7–25.2)	0.1 (– 0.6 to – 0.7)
High-income Asia Pacific	62,652.3 (52,382.8–74,360.9)	78,997.7 (68,905.1–90,260.6)	32.9 (27.6–39.1)	31.5 (27.3–36.5)	– 4.3 (– 9.0 to – 1.3)
High-income North America	200,836.4 (173,238.5–232,436.8)	257,777.8 (236,252.7–284,187.5)	62.4 (53.7–72.0)	52.0 (47.5–56.9)	– 16.6 (– 22.3 to – 9.9)
North Africa and Middle East	61,824.3 (50,782.1–75,020.1)	140,637.9 (117,038.5–168,090.2)	26.7 (22.3–31.5)	26.6 (22.5–31.2)	– 0.2 (– 1.7 to – 1.6)
Oceania	1078.6 (884.9–1322.0)	2385.6 (1934.6–2925.5)	24.9 (20.7–29.6)	24.1 (20–28.7)	– 3.0 (– 5.0 to – 0.8)
South Asia	328,618.8 (267,223.4–401,086.7)	743,524.0 (611,176.0–901,906.4)	38.1 (31.6–45.9)	43.0 (35.9–51.7)	12.8 (10.9–14.9)
Southeast Asia	93,540.7 (76,473.3–113,689.3)	174,246.5 (143,846.9–208,675.3)	26.0 (21.6–30.9)	25.3 (21.2–30.1)	– 2.6 (– 3.9 to – 1.5)
Southern Latin America	15,849.3 (13,664.8–18,265.2)	24,167.2 (20,962.5–27,835.3)	33.6 (28.9–38.6)	31.6 (27.4–36.6)	– 5.8 (– 7.9 to – 3.9)
Southern Sub-Saharan Africa	8604.9 (7042.0–10,531.2)	15,207.3 (12,560.8–18,528.5)	22.1 (18.5–26.4)	21.7 (18.1–25.9)	– 5.8 (– 7.9 to – 3.9)
Tropical Latin America	23,704.1 (20,682.2–27,345.5)	47,509.1 (41,862.2–53,770.0)	20.1 (17.7–22.7)	19.4 (17.1–21.9)	– 3.4 (– 4.7 to – 2.0)
Western Europe	123,270.9 (107,562.6–140,276.4)	170,344.2 (149,047.3–194,756.8)	25.4 (22.1–29.2)	26.3 (22.8–30.1)	3.5 (1.5–5.3)
Western Sub-Saharan Africa	33,289.8 (27,664.2–40,190.6)	82,116.6 (68,165.6–98,901.6)	26.0 (22.0–30.6)	26.7 (22.7–31.4)	3.0 (2.5–3.5)

EAPC, estimated annual percentage change; UI, uncertainty interval

In the SDI region level, the two highest quintiles of SDI regions had the highest acute pancreatitis burden in 2019, with ASIR of 38.1 and 36.7 per 100,000 respectively (Table 1). Subgroup analysis by geographical zone showed that Eastern Europe and High-income North America had the highest ASIR in both 1990 and 2019 (Eastern Europe: 71.2 in 1990 and 79.6 in 2019 per 100,000; High-income North America: 62.4 in 1990 and 52.0 in 2019 per 100,000). Most of the regions (13/21, 61.9%) observed a steadily annual decrease in ASIR during the last 30 years. However, South Asia and Eastern Europe showed a mushrooming rise in ASIR (EAPC of South Asia: 12.8%, 95% UI 10.9–14.9%; EAPC of Eastern Europe: 11.7%, 95% UI 10.8–12.7%) (Table 1).

**Fig. 2:**
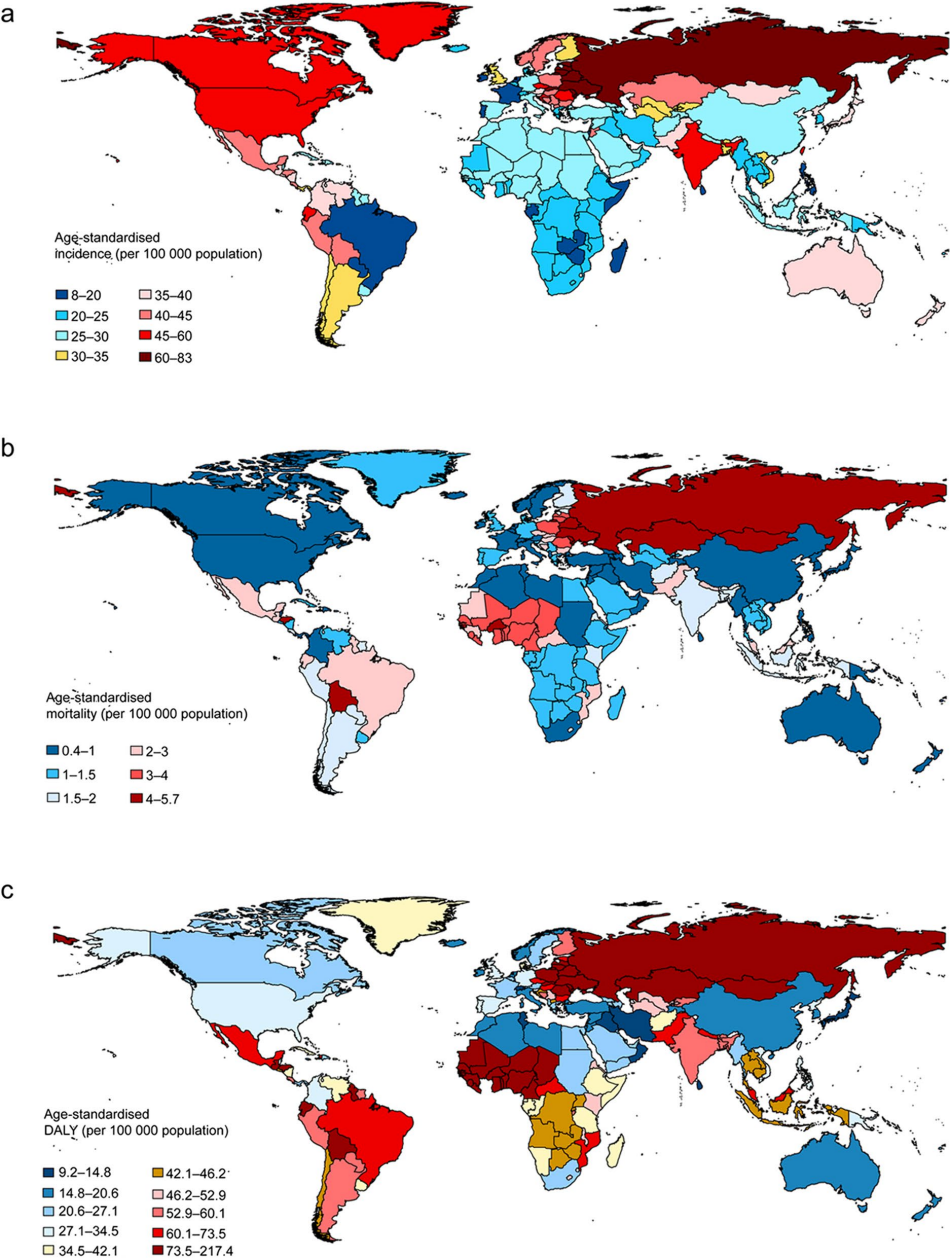
The age-standardized incidence (**a**), mortality (**b**) and DALY rates (**c**) of acute pancreatitis per 100,000 person-years by location for both sexes combined, 2019. DALY: disability-adjusted life-year

In 2019, countries with the greatest number of incident cases of acute pancreatitis were India (618,862.3), China (493,765.4), and USA (228,699.2) (Additional file 1: Table S1). The highest ASIR (more than 60 cases per 100,000 population) were observed in the Russia (82.0/100,000), Ukraine (77.0/100,000), Republic of Moldova (71.3/100,000), Belarus (69.7/100,000), Slovakia (68.4/100,000), Lithuania (64.8/100,000), Estonia (62.8/100,000) and Latvia (61.7/100,000) (Fig. 2a, Additional file 1: Table S1).

### Mortality due to acute pancreatitis

Globally, there were 69,817.6 (95% UI 62,046.7–82,529.3) deaths in 1990 and 115,053.2 (95% UI 104,304.4–128,173.4) deaths in 2019 caused by acute pancreatitis, increasing by 64.8% (95% UI 55.3–68.1%). However, the global ASMR decreased from 1.7 deaths per 100,000 (95% UI 1.5–2.0) in 1990 to 1.4 (95% UI 1.3–1.6) deaths per 100,000 in 2019, with EAPC of − 17.2% (95% UI − 27.1% to − 6.6%) (Table 2). The ASMR in males was much higher than that in females (2.2 vs 1.3 per 100,000 in 1990; 1.9 vs 1.0 per 100,000 in 2019) (Fig. 1 b).

**Table 2:** The death of acute pancreatitis in 1990/2019 and temporal trends

	Death cases (95% UI)		Age-standard mortality rate (per 100,000)		EAPC of mortality rate
	1990	2019	1990	2019	1990–2019 (%)
Global	69,817.6 (62,046.7–82,529.3)	115,053.2 (104,304.4–128,173.4)	1.7 (1.5–2.0)	1.4 (1.3–1.6)	− 17.2 (− 27.1 to − 6.6)
Sex					
Male	43,129.0 (37,615.8–51,561.2)	71,983.2 (63,882.3–81,418.6)	2.2 (1.9–2.6)	1.9 (1.7–2.1)	− 14.5 (− 24.4 to − 3.1)
Female	26,688.6(23,528.9–32,952.8)	43,070 (36,592.6–50,773.7)	1.3 (1.1–1.6)	1.0 (0.8–1.2)	− 21.5 (− 36.2 to − 8.3)
SDI factor					
High SDI	12,159.0 (11,441.0–13,388.5)	16,160.0 (14,600.9–18,476.3)	1.2 (1.1–1.3)	0.9 (0.8–1.0)	− 26.9 (− 30.9 to − 21.5)
High-middle SDI	21,366.4 (19,824.1–25,093.1)	34,393.0 (31,194.4–37,307.4)	2.0 (1.9–2.4)	1.8 (1.6–1.9)	− 11.7(− 27.4 to − 3.0)
Middle SDI	15,561.3 (13,213.7–20,288.1)	25,776.2 (22,835.9–30,111.2)	1.4 (1.2–1.9)	1.1 (1.0–1.3)	− 24.7 (− 39.6 to − 7.6)
Low-middle SDI	14,314.8 (11,855.7–18,507.1)	26,440.8 (21,686.3–30,710.8)	2.2 (1.8–2.8)	1.9 (1.5–2.2)	− 13.7 (− 31.8 to − 13.1)
Low SDI	6385.7 (4653.4–8612.9)	12,232.2 (9722.6–15,560.5)	2.4 (1.7–3.3)	2.1 (1.7–2.7)	− 13.5 (− 27.8 to − 4.8)
Region					
Andean Latin America	1076.8 (811.2–1299.4)	1444.9 (1122.3–1971.2)	4.6(3.4–5.6)	2.5 (2.0–3.5)	− 44.2 (− 59.6 to − 15.1)
Australasia	215.9 (198.6–233.7)	339 (292.5–396.5)	0.9 (0.9–1)	0.7 (0.6–0.8)	− 29.7 (− 37.7 to − 17.5)
Caribbean	384.3 (338.7–436.9)	659.0(557.6–795.7)	1.4 (1.3–1.6)	1.3 (1.1–1.6)	− 9.6 (− 24.2 to 7.0)
Central Asia	1179.1 (1016.3–1321.5)	1653.3 (1381.5–1884.1)	2.4 (2.1–2.8)	2.1 (1.8–2.4)	− 12.1 (− 24.3 to 1.7)
Central Europe	4476.7 (4274.1–5033.9)	5140.4 (4513.4–5828.0)	3.2 (3.1–3.6)	2.7 (2.4–3.1)	− 15.6 (− 27.2 to − 4.2)
Central Latin America	2018.5 (1909.5–2130.6)	4332.6 (3728.8–5065.2)	2.1 (1.9–2.2)	1.8 (1.6–2.1)	− 12.4 (− 24.8 to 2.2)
Central Sub-Saharan Africa	452.4 (302.2–763.6)	911.5 (530.4–1648.7)	1.8 (1.2–3.1)	1.4 (0.8–2.7)	− 19.7 (v43 to 4.6)
East Asia	9323.9 (7503.7–12,418.1)	11,289.8 (8798.1–13,450.7)	1.1 (0.9–1.5)	0.6 (0.5–0.7)	− 44.3 (− 60.6 to − 26.3)
Eastern Europe	7662.1 (6921.7–10,789)	15,578.4 (13,366.9–17,734.7)	2.9 (2.6–4.1)	5.3 (4.5–6)	83.2(20.1–115.4)
Eastern Sub-Saharan Africa	1398.2(899.1–2104.9)	2769.3 (1710.4–4739.2)	1.7 (1.0–2.6)	1.5 (0.9–2.7)	− 12.1 (− 34.6 to 9.5)
High-income Asia Pacific	1792.4 (1602.4–2057.1)	2248.0 (1886.8–2803.8)	1.0 (0.9–1.1)	0.5 (0.4–0.6)	− 49.5 (− 55.7 to − 39.5)
High-income North America	3348.2 (3123.7–3579.4)	5443.7 (4993.9–5955.3)	1.0 (0.9–1)	0.9 (0.9–1.0)	− 5.6 (− 9.6 to 1.3)
North Africa and Middle East	1718.7 (1389–2350.2)	3394.9 (2654.5–4069.2)	1.1 (0.9–1.5)	0.9 (0.7.0–1.1)	− 21.2 (− 42.6 to 2.9)
Oceania	36.8 (25.7–52.2)	76.7 (53.7–107.9)	1.1 (0.7–1.5)	0.9 (0.7–1.3)	− 11.9 (− 32 to 11)
South Asia	14,050.3 (11,344.3–19,215)	25,936.8 (20,085.4–31,351.7)	2.3 (1.8–3.1)	1.8 (1.4–2.2)	− 20.2 (− 42.4 to 11.9)
Southeast Asia	4993.4 (3842.9–7457.8)	7913.5 (6540.7–11,170.8)	1.8 (1.4–2.6)	1.3 (1.1–1.8)	− 25.1 (− 40.9 to − 2.5)
Southern Latin America	1267.6 (1136.0–1374.1)	1495.7 (1347.0–1732.5)	2.8 (2.5–3.0)	1.8 (1.7–2.1)	− 33.8 (− 42.5 to − 17.9)
Southern Sub-Saharan Africa	325.4 (263.3–414.7)	573.9 (453–675.4)	1.0 (0.8–1.3)	0.9 (0.7–1.1)	− 7.8 (− 33.5 to 10.6)
Tropical Latin America	2329.6(2212.2–2485.9)	5557.4 (4793.8–5987.2)	2.3 (2.1–2.4)	2.3 (2.0–2.5)	1.0 (− 15.1 to 12)
Western Europe	7986.7 (7447.9–8873.5)	9984.5 (8925.7–11,455.5)	1.4 (1.4–1.6)	1.1 (1.0–1.2)	− 25.7 (− 30.9 to − 19.4)
Western Sub-Saharan Africa	3781.0 (2498.4–5769.9)	8310.1 (5927.1–11,883.0)	3.8 (2.5–5.8)	3.6 (2.7–5.1)	− 3.9 (− 30.4 to 28.5)

EAPC, estimated annual percentage change; UI, uncertainty interval

Subgroup analysis by SDI indicated that the high-middle SDI region had the most deaths in both 1990 (21,366.4) and 2019 (34,393.0). As for a specific geographical zone, South Asia, Eastern Europe and East Asia were the top 3 regions with the most acute pancreatitis-related deaths in 2019 (Table 2). India, Russia and China were the top 3 countries that had the most deaths in 2019, with 20,455.9, 11,615.3 and 10,663.6 deaths respectively (Additional file 1: Table S1). Russia also had the highest ASMR in 2019, with 5.7 (95% UI 4.8–6.7) deaths per 100,000. Even though had a relatively large number of death cases, China had a very low ASMR in 2019, with 0.6 deaths per 100,000 and ranking eleventh from the bottom (Fig. 2b). The top five countries with the highest ASMR in 2019 were Russia (5.7/100,000), Kazakhstan (5.0/100,000), Guinea-Bissau (4.8/100,000), Ukraine (4.7/100,000), and Burkina Faso (4.6/100,000) (Additional file 1: Table S1).

### Summary measures of health by DALYs

The number of DALYs increased from 2,437,815.7 in 1990 to 3,641,105.7 in 2019 in the globe, but the age-standardized DALY rate improved from 59.3 in 1990 to 44.4 in 2019 per 100,000, with EAPC of − 17.6% (95% UI − 27.0% to − 7.8%) (Table 3). Males were the main contributor to the age-standardized DALY rate compared with females (Fig. 1c). Subgroup analysis by socio-demographic factor demonstrated that although the high-middle SDI region had the most DALYs from 1990 to 2019 (720,516.0 in 1990 and 1,057,814.6 in 2019), the age-standardized DALY rate was highest in the low SDI region.

**Table 3:** The DALYs of acute pancreatitis in 1990/2019 and temporal trends

	DALYs (95% UI)		Age-standardized DALY rate (per 100,000)		EAPC of DALY
	1990	2019	1990	2019	1990–2019 (%)
Global	2,437,815.7 (2,179,992.9–2,885,021.2)	3,641,105.7 (3,282,952.5–4,026,948.1)	53.9 (48.2–63.3)	44.4 (40.1–49.1)	− 17.6 (− 27.0 to − 7.8)
Male	1,633,012.8 (1,426,941.6–1,942,851.5)	2,502,438.2 (2,224,864.1–842,383.8)	72.4 (63.4–85.9)	62.0 (55.2–70.4)	− 14.4 (− 23.8 to − 3.4)
Female	804,802.9 (691,735.0–1,038,584.8)	1,138,667.5 (969,571.1–1,334,422.8)	35.2 (30.6–44.8)	27.0 (22.9–31.5)	− 23.5 (− 38.2 to − 11.9)
SDI factor					
High SDI	341,420.8 (321,211.0–370,387.3)	389,932.5 (360,177.7–429,354.3)	35.3 (33.2–38.3)	25.7 (23.8–28.4)	− 27.1 (− 31.8 to − 22.7)
High-middle SDI	720,516.0 (667,787.2–839,145.4)	1,057,814.6 (962,196.3–1,156,821.3)	63.7 (59.1–74.2)	57.1 (51.9–62.4)	− 10.5 (− 24.7 to − 1.7)
Middle SDI	580,739.7 (498,484.7–739,252.5)	813,217.3 (727,039.6–960,672.8)	43.7 (37.3–56.4)	31.6 (28.2–37.2)	− 27.8 (− 40.3 to − 14.2)
Low-middle SDI	549,795.0(462,013.9–726,880.2)	919,232.5 (751,488.8–1,066,251.6)	68.4 (57.0–88.3)	58.0(47.3–66.8)	− 15.3 (− 32.5 to 8.6)
Low SDI	244,325.0 (185,104.9–330,618.6)	459,380.2 (367,333.8–583,539.5)	75.1 (55.2–00.8)	64.0 (50.8–81.3)	− 14.9 (− 29.9 to 3.3)
Region					
Andean Latin America	41,681.2 (49,404.7–30,752.3)	43,654.9 (34,042.4–57,110.3)	144.8 (108.3–173.2)	72.5 (56.6–96.0)	− 49.9 (− 63.0 to − 25.6)
Australasia	5562.6 (6030.6–5108.7)	7137.4 (6312.5–8100.2)	24.4 (22.4–26.5)	16.6 (14.7–18.8)	− 32.2 (− 39.4.2 to − 21.6)
Caribbean	13,236.4 (15,634.5–11,659.6)	20,254.6 (16,880.2–24,620.4)	44.5 (39.3–51.6)	40.1 (33.3–48.7)	− 9.9 (− 23.8.0 to 5.7)
Central Asia	41,215.8 (45,305.5–36,642.2)	60,311.2 (51,431.9–69,088.5)	77.1 (68.3–85.3)	67.4 (57–76.9)	− 12.5 (− 24.4 to 1.0)
Central Europe	147,739.5 (161,428.9–141,054.8)	140,578.0 (123,451.4–158,896)	106.0 (101.2–115.9)	84.4 (74–95.6)	− 20.4 (− 30.9.0 to − 9.5)
Central Latin America	80,017.9 (84,085.9–74,360)	141,733.4 (121,885.5–165,140.4)	67.0 (63.3–70.8)	56.5 (48.7–65.7)	− 15.7 (− 27.4.0 to − 0.9)
Central Sub-Saharan Africa	17,413.0 (29,045.2–11,986.3)	35,823.5 (21,454.6–62,656.8)	54.4 (36.7–90.9)	44.6 (26.5–80.0)	− 18.0 (− 42.1.1 to 10.6)
East Asia	333,276.6(431,225.3–266,027.9)	319,973 (255,865.5–381,413.7)	31.7 (25.7–41.5)	16.5 (13.2–19.6)	− 47.9 (− 62.1.3 to − 32.1)
Eastern Europe	283,386.3 (383,366.6–254,907.8)	558,129.2 (481,178.6–640,436.3)	109.2 (98.2–146.8)	206.7 (178.3–237.9)	− 89.2 (− 29.5.2 to 120.3)
Eastern Sub-Saharan Africa	51,548.1 (78,409.9–34,736.6)	103,187.0 (65,327.8–170,638.8)	49.6 (32.1–74.6)	43.3 (27.1–73.7)	− 12.7 (− 34.4.1 to 14.2)
High-income Asia Pacific	54,433.0 (66,219.4–47,665.2)	48,064.3 (41,966.4–56,862.1)	27.6 (24.1–33.4)	14.7 (12.7–17.4)	− 46.6 (− 57.3.3 to − 37)
High-income North America	100,034.4 (109,713.5–92,703.1)	145,495.4 (134,413.4–161,035.3)	30.8(28.5–33.9)	28.6 (26.4–31.6)	− 7.4 (− 10.7.0 to − 1.9)
North Africa and Middle East	50,486.0 (69,094.5–42,546.5)	91,833.5 (74,033.1–111,078.4)	25.8 (21.5–35.0)	19.5 (15.7–23.3)	− 24.6 (− 41.2.0 to − 4.6)
Oceania	1540.1 (2186.2–1100.1)	3181.1 (2194.1–4459.1)	33.9 (23.9–47.8)	30.3 (21.4–42.0)	− 10.7 (− 32.7.1 to 16.6)
South Asia	548,424.8 (759,731.1–448,431.7)	909,993.5 (707,700.0–1,093,715.6)	69.9 (56.5–95.3)	55.3 (43.1–66.4)	− 20.9 (− 43.6.0 to 8.2)
Southeast Asia	187,984.2 (286,963–145,388)	254,592.2 (204,677.0–376,978.7)	54.5 (42.3–81.1)	37.9 (30.8–54.8)	− 30.5 (− 43.6.1 to − 12.7)
Southern Latin America	37,055.6 (40,031.4–33,771.4)	40,292.4 (36,539.8–45,858.7)	78.6 (71.7–85.0)	51.6 (46.8–58.8)	− 34.3 (− 42.4.2 to − 21.1)
Southern Sub-Saharan Africa	12,983.4 (16,365.4–10,471.3)	21,220.6 (16,850.1–25,384.5)	34.6 (28.1–43.7)	30.0 (23.8–35.7)	− 13.3 (− 38.3.0 to 5.8)
Tropical Latin America	90,244.6 (95,308.1–85,172.8)	175,860.2 (155,755.4–189,220.5)	75.4 (71.0–79.9)	70.6 (62.5–76.0)	− 6.3 (− 17.3.0 to 2.0)
Western Europe	196,013.5(212,945.9–184,807.6)	199,700.1 (183,864.2–224,326.9)	38.8 (36.6–41.9)	27.1 (25.2–30.4)	− 30.1 (− 35.2.2 to − 24.1)
Western Sub-Saharan Africa	143,538.8 (213,834.4–95,218.6)	320,090.3 (226,679.1–458,546.1)	122.5 (80.9–189.4)	115.5 (82.3–165.9)	− 5.7 (− 33.1 to 26.2)

EAPC, estimated annual percentage change; UI, uncertainty interval; DALY, disability-adjusted life-year

In the subgroup analysis by geographical zone, we found that Russia, Ukraine, and Republic of Moldova were the top 3 countries that had the highest age-standardized DALY rate in 2019 (with 217.3, 196.2 and 173.1 per 100,000 respectively) (Fig. 2c). Republic of Korea had the fastest decrease in age standard DALY rate during the past 30 years (dropped from 47.3 to 17.7 per 100,000, EAPC = − 62.6%, 95% UI − 79.9% to − 33.6%). By contrast, Russia showed a sharply increase in age-standardized DALY rate from 1990 to 2019, increased from 103.1 to 217.3 per 100,000 (EAPC = 110.9%, 95% UI 26.3–155.3%) (Additional file 1: Table S1).

### The correlation between SDI and the burden of acute pancreatitis

**Fig. 3:**
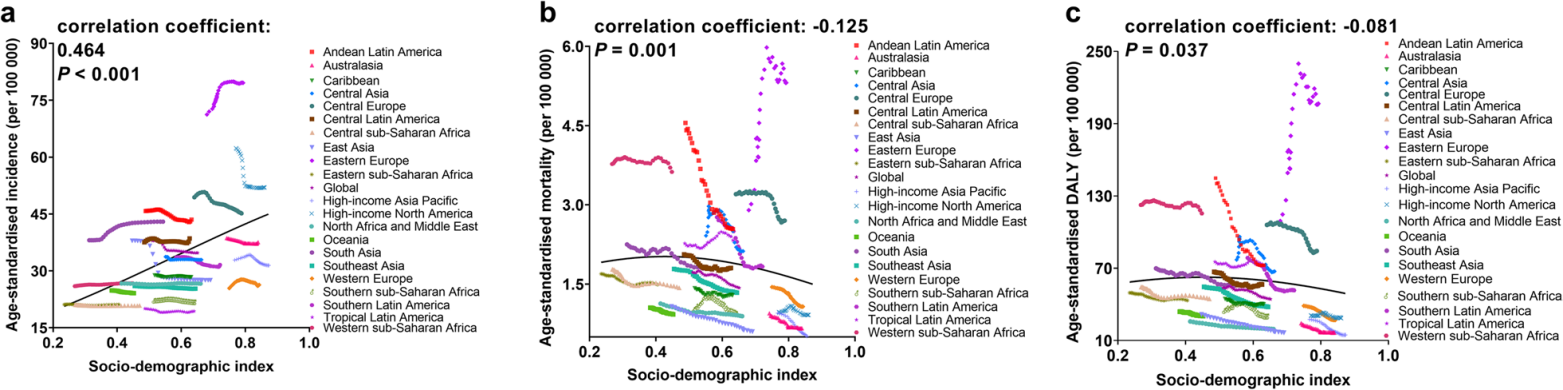
The change trends and correlation analyses of acute pancreatitis’ burden and SDI for 21 world regions from 1990 to 2019. The age-standardized incidence (**a**), mortality (**b**) and DALY (**c**) rates per 100,000 person-years is shown. The solid black line is a mixed-effects and spatiotemporal Gaussian process regression, and represents the expected values across the spectrum of the SDI. DALY: disability-adjusted life-year; SDI: socio-demographic index

We investigated the correlation between SDI and ASIR, ASMR, and age-standardized DALY rate in 21 regions around the globe from 1990 to 2019. The results revealed that the ASIR was positively correlated with SDI (*P* < 0.001). In the contrary, both the ASMR (*P* = 0.001) and age-standardized DALY rate (*P* = 0.037) were negatively correlated with SDI (Fig. 3). Notably, despite gains in SDI over time, Eastern Europe had much higher age-standardized incidence, mortality and DALY rates than expected values based on SDI for nearly all years between 1990 and 2019.

### The burden of acute pancreatitis and age structure

We analyzed the incidence rate in five age groups: under 5 years, 5–14 years, 15–49 years, 50–69 years, and over 70 years in the globe and different SDI regions. The results demonstrated that the incidence rate (per 100,000) in people above 70 years was the highest across all regions from 1990 to 2019 (Additional file 1: Fig. S1). Based on data of 1990 and 2019, all the incidence, mortality and DALY rates rose with advancing age; of note, the mortality rate rose sharply in patients aged 70 years and older (Additional file 1: Fig. S2).

### The mortality of acute pancreatitis due to alcohol etiology

In 1990, there were 0.8 males died per 100,000 due to alcohol-related pancreatitis in the globe, accounted for 36.1% (0.8/2.1) of all cause pancreatitis-related ASMR; among females, this percentage was 15.4% (0.2/1.3) (Additional file 1: Fig. S3a). The attributable fraction of alcohol etiology on mortality was highest among people in the high SDI region for both males and females.

Up to 2019, alcohol etiology still responsible for a sizable fraction of mortality rate in males than that in females (40.2% vs 12.3%) globally, and was the dominant cause for acute pancreatitis’ ASMR in males in the high (50.2%) and high-middle SDI (52.4%) regions (Additional file 1: Fig. S3b). The five highest alcohol etiology caused ASMR were seen in Russia (2.7/100,000), Ukraine (2.3/100,000), Republic of Moldova (2.3/100,000), Belarus (2.2/100,000) and Lithuania (2.1/100,000) (Additional file 1: Fig. S4).

## Discussion

This study provides a systematic analysis of acute pancreatitis’ incidence, mortality, DALY and corresponding trends across all 204 countries and territories over a 30-year period. We estimated that in 2019, 2,814,972.3 acute pancreatitis occurred, with 115,053.2 (4.1%) person died globally. Between 1990 and 2019, the age-standardized incidence of acute pancreatitis declined in most countries, but the condition persisted severe in some of the regions, especially in Eastern Europe and High-income North America.

Our global estimate of acute pancreatitis’ incidence in 2019 is close to the estimation in previous meta-analysis (33.7/100,000); in addition, the GBD estimation of 1.4 deaths per 100,000 is also similar to the meta-analytical result (1.6 deaths per 100,000) [7]. Of note, the previous analyses failed to make the combined best use of different types of data on incidence, mortality, DALY, and risk factors reported in literature sources, claims databases, and vital registration systems. GBD estimates the attributable disease burden using a comparative risk assessment strategy through all countries and territories around the world [26]. The global analysis and cross-region comparisons could improve the comprehensive understanding of the burden of acute pancreatitis.

Although our results revealed an increase in acute pancreatitis’ incident cases during the 30-year study period, the mortality rate declined continuously. We have also observed a decreasing mortality rate over the last decade [8, 27–30]. These outcomes are justifiable, for patients with acute pancreatitis are becoming easier to identify with better testing approaches, and the complications can be detected at an earlier stage in the disease course [31]. Furthermore, improvements on the treatment of severe cases in intensive care units, combined with multidisciplinary management strategies have all contributed to the decrease in the acute pancreatitis-related death [32, 33].

However, it should be pointed out that patients in low SDI region had more than twofold higher mortality of acute pancreatitis than people in the high SDI region. Socioeconomic factors are important variables in acute pancreatitis’ epidemiology, especially for the mortality. There were indications that the size of hospital was closely associated with the risk of acute pancreatitis-related fatality. Compared with small hospitals, patients admitted into the large hospitals had a lower risk of death [34–36]. Better outcomes have also been observed for hospitals and surgeons with high volumes of cases [37, 38]. By contrast, the prognosis of acute pancreatitis in low-income regions or socially deprived areas was poorer [39, 40]. The morbidity and mortality of acute pancreatitis would be expected to correlate with the national health system infrastructure as regards the existence or not of specialist tertiary pancreatitis units, and improved outcomes might be achieved in countries with improved access to clinical resources such as specialist tertiary pancreatitis services.

Differences also existed between different age groups. We found that the incidence rate was significantly higher in population over 70 years old. Actually, aging is an important factor that contributing to acute pancreatitis. The incidence of acute pancreatitis attributable to gallstone increased sharply with age for both men and women [40]. In addition, Floyd et al. [13] found that the increase in the drug consumption, such as azathioprine, was also correlated with the higher incidence of acute pancreatitis in the elderly. Moreover, strong association between old age and mortality rate was also reported previously [13], and this was in line with our finding that mortality rate increased with aging and rose sharply in those aged 70 years and older. This is to be expected in relation to the more numerous and more severe coexisting conditions in the elderly people [41, 42].

In GBD 2019, we found alcohol etiology was an important risk factor for acute pancreatitis-related death. Males were much more likely to suffer from fatality due to alcohol-induced acute pancreatitis. In pace with the drinking prevalence increased towards higher levels of SDI [43], the fraction of mortality attributed to alcoholic acute pancreatitis also had a mushrooming rise towards high SDI region for both men and women. Notably, there were evidence indicated that the type of alcohol consumption would affect the acute pancreatitis’ risk. Roberts et al. [40] revealed that alcoholic acute pancreatitis was positively correlated with spirits and beer, but negatively with wine. Other studies showed that the risk of acute pancreatitis had a dose–response association with the number of units of spirits consumption, but not associated with beer or wine [44]. Therefore, public health policies that focus on reducing population-level alcohol intake might be effective in reducing the morbidity and mortality of acute pancreatitis.

This study has some limitations. First, apart from alcohol etiology, gallstone is another major cause of acute pancreatitis in most countries [45], so are the other less common but meaningful causes such as hypertriglyceridemia. However, the quantitative effect of gallstone and other causes on acute pancreatitis could not be assessed owing to unavailable data in the current round of GBD. Second, in order to formulate more effective preventive measures, proper stratification of severity grades and analysis for subtypes of acute pancreatitis according to aetiology is necessary. However, based on the data of GBD 2019, we couldn’t obtain such information. In addition, GBD faces several challenges for estimating cause-specific non-fatal and fatal burden of acute pancreatitis. Even though it employs spatiotemporal methods to inform estimates for locations with sparse data, these strategies cannot completely resolve issues when data are not available for some regions. As vital statistic systems and civil registration provide important information for disease preventions and public health policies, strengthening of these systems is essential for public health. An ongoing effort of adding new sources of data to the GBD should be taken to overcome the limitations.

## Conclusions

In summary, the age-standardized incidence, mortality and DALY rates of acute pancreatitis decreased gradually in the globe. However, the overall burden remains high with aging population, and will not decrease without effective strategies to address associated risk factors. The attributable burden is higher in males and elderly, and alcohol etiology is an important driver that contribute to the morbidity and mortality. There were substantial differences in the burden of acute pancreatitis across regions, geographically targeted considerations are needed to tailor future interventions to relieve the burden of acute pancreatitis in specific countries, especially for Eastern Europe.

## Supplementary Information

**Additional file 1.** Supplementary methods, tables, and figures.

## Data Availability

The datasets generated for this study can be found in the GBD at http://ghdx.healthdata.org/gbd-results-tool.

## Abbreviations

CODEm: Cause of death ensemble model
DALYs: Disability-adjusted life year
GBD: Global burden of diseases
EAPC: Estimated annual percentage change
ASIR: Age-standardized incidence rate
ASMR: Age-standardized mortality rate
GHDx: Global health data exchange
ICD: International classification of diseases
SDI: Socio-demographic Index
ST-GPR: Spatiotemporal Gaussian process regression
UIs: Uncertainty intervals
YLDs: Years lived with disability
YLLs: Years of life lost
