# Supplementary material for: The global, regional, and national burden of acute pancreatitis in 204 countries and territories, 1990–2019
Source: BMC Gastroenterol. 2021 Aug 25;21:332. doi: 10.1186/s12876-021-01906-2 (PMC8390209; doi:10.1186/s12876-021-01906-2)
Supplement: Supplementary file 1 — Additional file 1. Supplementary methods, tables, and figures. [file 12876_2021_1906_MOESM1_ESM.docx]

**Supplementary**

**Supplementary methods**

**
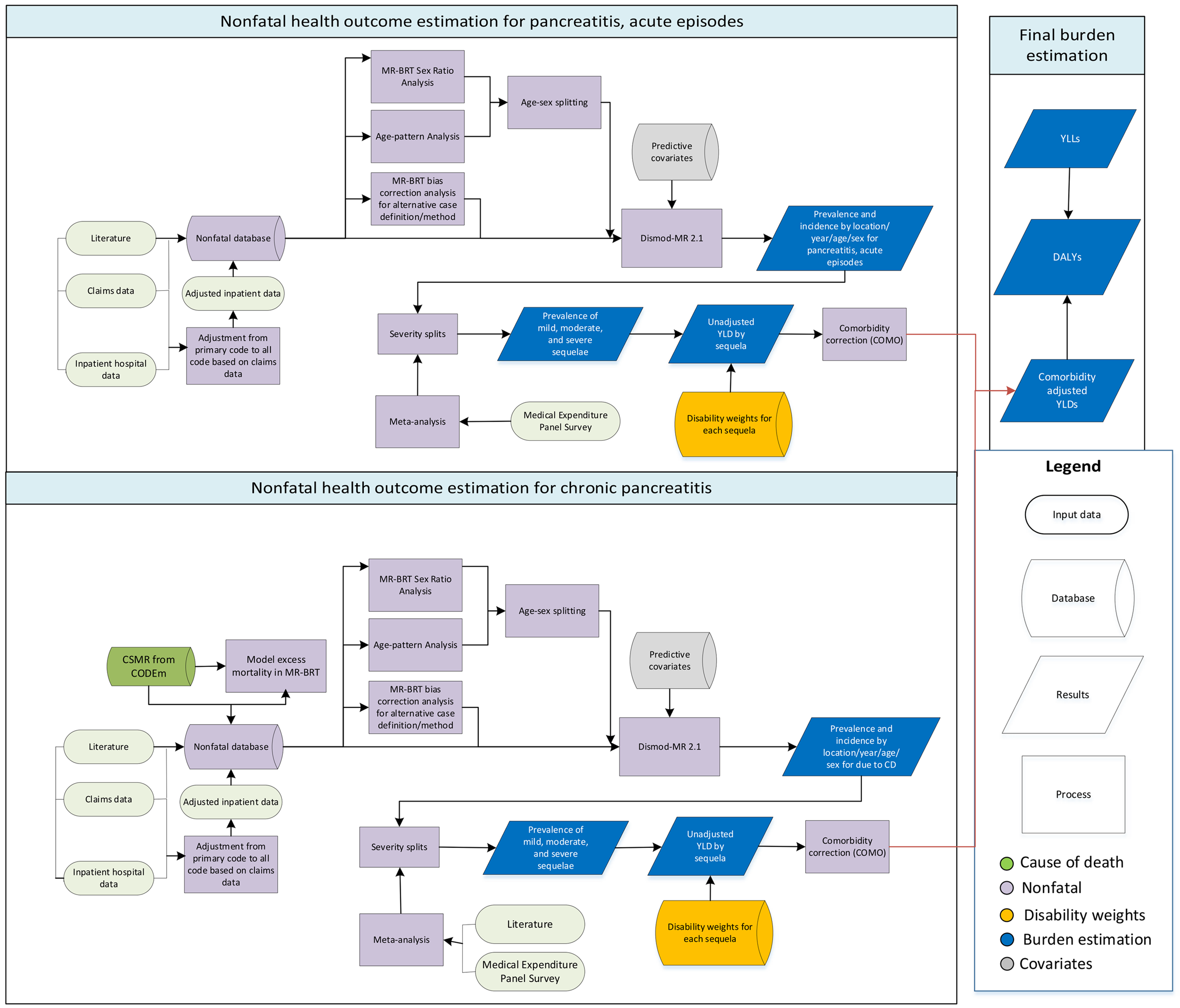
Flowchart for pancreatitis**

**Case definition**

Pancreatitis is the inflammation of the pancreas. Acute pancreatitis involves active inflammation and injury to the pancreas, resulting in severe upper abdominal pain and nausea, inappropriate release of pancreatic contents, and a systemic inflammatory response with fever, low blood pressure, and, in some cases, failure of one or more organs. Chronic pancreatitis involves permanent damage to the pancreas from longstanding or recurrent inflammation; this produces chronic or episodic abdominal pain and nausea and ultimately failure of the pancreas to produce and release digestive enzymes and hormones, leading to chronic diarrhea, poor absorption of nutrients from food, and diabetes. Individuals with chronic pancreatitis can have superimposed episodes of acute pancreatitis. In prior rounds of GBD, we modelled acute and chronic pancreatitis together, but in GBD 2019 we developed separate models for these two diseases.

ICD10 codes are K85 for acute and K86 for chronic pancreatitis. ICD9 code 577.0 corresponds to acute pancreatitis, and 577 and the remainder of its four-digit and five-digit constituents refer to chronic or unspecified pancreatitis.

**Input data**

Studies were added to the acute database if they measured the incidence of acute pancreatitis as defined by appropriate ICD codes, or by a combination of clinical, biochemical, and radiographic criteria. The acute database included studies that measured incidence of first episode of acute pancreatitis only, and studies that measured incidence of all acute pancreatitis, including recurrent episodes. Studies that included individuals with underlying chronic pancreatitis were excluded from the acute database.

Studies were added to the chronic database if they employed appropriate ICD codes or appropriate clinical, biochemical, and radiographic criteria of chronic pancreatitis. Some studies reported incidence of acute and chronic disease separately and data were extracted to both databases, but those few studies that reported only a single measure for both disorders were excluded.

**Data processing**

Hospital discharge data provide observations about encounters, generally with only the primary diagnostic code for the encounter. Claims data, on the other hand, link claims for all inpatient and outpatient encounters for a single individual, and provide primary and secondary diagnoses for all encounters.

Similar to GBD 2017, in the acute database, an individual was extracted from claims data as an incident case if that individual had one or more inpatient encounters with an appropriate ICD code as any diagnosis; readmissions within 30 days were assumed to be for the same episodes of illness. Hospital discharges were included only if the primary discharge diagnosis was a code for acute pancreatitis, and incident cases were estimated from number of discharges using a correction factor from claims data.

In the chronic database, claims data linked multiple inpatient and outpatient claims to a single individual; prevalent cases were extracted if an individual had at least one inpatient or two outpatient encounters with a chronic pancreatitis ICD code as any diagnosis. Data from hospital discharges were, then, adjusted using correction factors from claims, converting encounters to estimates of cases, accounting for most locations providing only primary diagnostic codes, and estimating outpatient cases from inpatient cases. Encounter data from outpatient facilities used in GBD 2017 were excluded in GBD 2019 because they were highly heterogeneous and inconsistent with other data sources from the same locations.

In GBD 2019, we improved the bias adjustment methods to allow a more direct comparison between different case definitions and/or study designs. In GBD 2017, we used data from published studies that employed rigorous case definitions as our reference standard for acute pancreatitis and adjusted clinical administrative data toward this reference standard by marking administrative data with binary covariates, and estimating a fixed effect for this covariate in our DisMod meta-regression modeling process. This amounts to adjusting data using an ecological comparison, and vulnerable to compositional bias; if data from different location-years were collected using different methods or case definitions, true spatiotemporal differences in epidemiology can be erroneously adjusted, and differences truly due to differences in methods can be erroneously estimated as differences in underlying epidemiology.

In GBD 2019, we avoided this risk by making pre-modeling bias adjustments and dropping data types that could not be rigorously adjusted. This was done by conducting a meta-regression of the relationship between data points matched on year, age, sex, and location, but differing with regard to one or more study design characteristic.

Like in GBD 2017, we decided to use data from literature studies that identified cases through detailed chart review as the reference standard for the acute pancreatitis model. These studies used a combination of clinical presentation, biochemical, and radiographic findings to validate a case definition, which we refer to as “stringent criteria” in shorthand. Using the stringent criteria, we would, then, adjust other ICD-code based administrative data without validation (i.e. data from claims and hospital discharges). However, the number of matched pairs between reference and alternative (based on year, age, sex and location) was small and yielded highly uncertain adjustment factors for the alternative case definitions. As a result, a new case definition was adopted in GBD 2019: diagnosis of acute pancreatitis as indicated by ICD code in a clinical encounter. Other case definitions and study design characteristics were adjusted toward this new reference standard.

The chronic pancreatitis model used ICD-code based administrative data as the reference standard in GBD 2017 due to scant literature data that were available. In GBD 2019, we attempted to employ the new bias adjustment method for chronic pancreatitis using the more rigorous case definition based on clinical, biochemical, and radiographic findings, but, like in the acute pancreatitis model, we could not find an adequate number of comparison pairs to inform reliable adjustment factors. Therefore, we decided to use the same ICD-based administrative data as the reference standard in GBD 2019, adjusting other case definitions and study design characteristics to this reference standard.

For both acute and chronic pancreatitis models, the USA claims data from the year 2000 and from the years 2010–2016 were each adjusted to the reference to adjust for selection bias due to commercial insurance.

**Modeling strategy**

Similar to GBD 2017, we ran a DisMod-MR 2.1 model to produce estimates by age, sex, year, and country, and no other significant modeling changes were made in GBD 2019. The prior value of remission was bounded from 8 to 9 (a duration from about six weeks) for all ages. The minimum coefficient of variation at the regional, super-regional, and global-level was changed from 0.4 to 0.8 in GBD 2019 to improve model fit against input data. Predictive covariates included were per capita alcohol consumption on incidence and healthcare access and quality index on excess mortality rate (EMR).

Betas and exponentiated values of predictive covariates (which can be interpreted as an odds ratio) are shown in the table below:

**Table.** Summary of covariates used in the acute pancreatitis DisMod-MR meta-regression model

| **Covariate** | **Type** | **Parameter** | **Exponentiated beta (95% Uncertainty Interval)** |
| --- | --- | --- | --- |
| Alcohol (litres per  capita) | Country-level | Incidence | 1.00 (1.00, 1.00) |
| Healthcare access and  quality index | Country-level | Excess mortality rate | 0.98 (0.15, 7.31) |

(Cited from Lancet. 2020 Oct 17;396(10258):1204-1222. doi: 10.1016/S0140-6736(20)30925-9. Supplement)

**Table S1** Incidence, deaths and DALYs for acute pancreatitis in 2019, and percentage change of age-standardized rates in 204 countries and territories, 1990–2019

|  | **Incidence (95% UI)** | | | **Deaths (95% UI)** | | | **DALY (95% UI)** | | |
| --- | --- | --- | --- | --- | --- | --- | --- | --- | --- |
| **Location** | Count, 2019 | ASIR, 2019 | EAPC between 1990 and 2019 (%) | Count, 2019 | ASMR, 2019 | EAPC between 1990 and 2019 (%) | Count, 2019 | Age-standardised DALY rate, 2019 | EAPC between 1990 and 2019 (%) |
| Afghanistan | 6202.6  (5045.5 to 7565.3) | 28.6  (24.0 to 33.5) | 0.0  (0.0 to 0.0) | 184.9 (124.7 to 286.9) | 1.6 (1.0 to 2.6) | 2019 (%) | 6275.4 (3924.5 to 9851.1) | 36.1 (24.5 to 56.1) | -0.2 (-0.4 to 0.1) |
| Albania | 1354.3  (1130.9 to 1597.2) | 39.3  (32.8 to 46.5) | 0.0  (-0.1 to 0.0) | 28.5 (19.7 to 42.1) | 0.7 (0.5 to 1.1) | -0.3 (-0.5 to 0.0) | 755.7 (534.4 to 1199.8) | 21.1 (15.1 to 33.9) | -0.3 (-0.5 to 0.0) |
| Algeria | 10190.8  (8377.0 to 12187.2) | 26.4  (21.9 to 31.2) | 0.0  (-0.1 to 0.0) | 207.8 (135.0 to 277.5) | 0.8 (0.5 to 1.0) | -0.3 (-0.5 to 0.1) | 5359.0 (3824.7 to 6907.3) | 15.4 (10.7 to 19.7) | -0.3 (-0.5 to 0.0) |
| American Samoa | 14.2  (11.8 to 16.8) | 28.1  (23.3 to 33.2) | 0.0  (0.0 to 0.0) | 0.8 (0.6 to 1.0) | 1.6 (1.2 to 2.0) | 0.1 (-0.3 to 0.6) | 26.5 (18.9 to 35.6) | 50.9 (36.5 to 68.5) | 0.1 (-0.3 to 0.8) |
| Andorra | 24.7  (20.4 to 29.3) | 21.0  (17.6 to 24.9) | -0.1  (-0.1 to -0.1) | 1.4 (0.9 to 2.1) | 0.9 (0.6 to 1.5) | -0.2 (-0.5 to 0.3) | 31.9 (19.9 to 49.4) | 24.6 (15.2 to 38.0) | -0.1 (-0.5 to 0.3) |
| Angola | 3959.3  (3203.9 to 4876.5) | 20.6  (17.2 to 24.5) | 0.0  (0.0 to 0.0) | 196.5 (118.2 to 325.6) | 1.5 (0.9 to 2.5) | -0.1 (-0.4 to 0.3) | 7713.0 (4669.3 to 12504.6) | 44.9 (27.4 to 73.9) | -0.2 (-0.4 to 0.3) |
| Antigua and Barbuda | 27.0  (22.2 to 32.3) | 27.1  (22.5 to 32.5) | 0.0  (-0.1 to 0.0) | 0.9 (0.7 to 1.1) | 0.9 (0.7 to 1.1) | -0.1 (-0.3 to 0.2) | 28.0 (23.0 to 33.8) | 27.1 (22.3 to 32.7) | -0.1 (-0.3 to 0.1) |
| Argentina | 15524.7  (13148.7 to 18264.2) | 30.9  (26.0 to 36.5) | -0.1  (-0.1 to -0.1) | 1041.5 (917.1 to 1260.6) | 2.0 (1.7 to 2.4) | -0.4 (-0.5 to -0.2) | 28615.5 (25281.3 to 33662.7) | 56.3 (49.7 to 65.8) | -0.3 (-0.4 to -0.2) |
| Armenia | 1111.6  (915.0 to 1325.0) | 30.0  (24.8 to 35.5) | 0.0  (0.0 to 0.0) | 31.2 (25.4 to 37.8) | 0.8 (0.7 to 1.0) | 0.3 (-0.1 to 0.6) | 862.8 (713.9 to 1027.5) | 23.1 (19.1 to 27.4) | 0.2 (-0.1 to 0.4) |
| Australia | 12151.2  (10240.7 to 14298.0) | 37.5  (31.4 to 44.4) | -0.1  (-0.1 to -0.1) | 279.9 (239.3 to 339.2) | 0.7 (0.6 to 0.8) | -0.3 (-0.4 to -0.2) | 5903.3 (5172.6 to 6877.2) | 16.3 (14.3 to 18.7) | -0.3 (-0.4 to -0.2) |
| Austria | 4985.1  (4587.4 to 5425.2) | 38.2  (35.2 to 41.7) | 0.0  (0.0 to 0.0) | 125.8 (108.2 to 170.5) | 0.7 (0.6 to 0.9) | -0.6 (-0.6 to -0.4) | 3051.2 (2650.5 to 3767.3) | 21.5 (18.6 to 26.0) | -0.6 (-0.6 to -0.4) |
| Azerbaijan | 3111.4  (2528.4 to 3744.7) | 28.5  (23.5 to 33.7) | 0.0  (0.0 to 0.0) | 64.9 (47.6 to 86.9) | 0.7 (0.5 to 1.0) | -0.1 (-0.4 to 0.5) | 2435.2 (1790.3 to 3196.0) | 22.4 (16.8 to 28.8) | -0.1 (-0.4 to 0.2) |
| Bahamas | 123.4  (102.0 to 146.6) | 30.4  (25.4 to 36.1) | 0.0  (-0.1 to 0.0) | 8.1 (6.4 to 10.4) | 2.0 (1.6 to 2.6) | -0.1 (-0.3 to 0.2) | 286.6 (223.1 to 370.6) | 67.5 (52.9 to 86.8) | -0.1 (-0.3 to 0.2) |
| Bahrain | 443.6  (350.5 to 542.6) | 29.4  (24.8 to 34.6) | -0.1  (-0.1 to -0.1) | 8.4 (5.3 to 11.1) | 1.4 (0.9 to 1.7) | -0.4 (-0.5 to -0.1) | 273.2 (166.7 to 364.2) | 25.4 (17.3 to 32.9) | -0.4 (-0.6 to -0.1) |
| Bangladesh | 49297.2  (40635.3 to 59626.1) | 32.1  (26.8 to 38.6) | 0.0  (0.0 to 0.0) | 2459.4 (1382.4 to 3753.4) | 1.8 (1.1 to 2.8) | -0.4 (-0.6 to -0.2) | 82793.6 (44301.1 to 130831.7) | 55.9 (30.8 to 86.9) | -0.4 (-0.7 to -0.2) |
| Barbados | 106.5  (88.2 to 125.3) | 28.0  (23.3 to 33.3) | 0.0  (-0.1 to 0.0) | 5.2 (4.1 to 6.6) | 1.2 (0.9 to 1.5) | 0.0 (-0.3 to 0.3) | 144.8 (112.7 to 180.2) | 35.7 (27.9 to 44.7) | -0.1 (-0.3 to 0.2) |
| Belarus | 8869.7  (7516.6 to 10295.7) | 69.7  (59.5 to 81.4) | 0.1  (0.0 to 0.1) | 585.8 (431.9 to 783.0) | 4.3 (3.1 to 5.8) | 0.4 (0.0 to 0.9) | 20179.9 (14781.1 to 27117.2) | 161.5 (117.9 to 215.3) | 0.5 (0.1 to 1.0) |
| Belgium | 3324.9  (2825.3 to 3843.8) | 20.9  (17.7 to 24.5) | -0.1  (-0.1 to -0.1) | 202.5 (173.3 to 251.4) | 0.9 (0.8 to 1.1) | -0.4 (-0.5 to -0.2) | 4308.6 (3750.0 to 5012.1) | 23.7 (20.5 to 27.6) | -0.4 (-0.5 to -0.2) |
| Belize | 99.5  (81.7 to 120.4) | 27.5  (22.8 to 32.8) | 0.0  (0.0 to 0.0) | 3.2 (2.7 to 3.9) | 1.0 (0.9 to 1.2) | 0.2 (-0.2 to 0.8) | 124.3 (102.8 to 151.1) | 34.5 (28.7 to 41.4) | 0.2 (-0.1 to 0.9) |
| Benin | 2005.8  (1661.0 to 2443.3) | 24.7  (21.1 to 29.0) | 0.0  (0.0 to 0.0) | 202.6 (143.7 to 288.8) | 3.3 (2.4 to 4.6) | 0.1 (-0.3 to 0.6) | 7909.7 (5443.9 to 11410.1) | 109.0 (77.2 to 155.7) | 0.1 (-0.3 to 0.7) |
| Bermuda | 26.4  (22.0 to 30.9) | 29.2  (24.4 to 35.1) | -0.1  (-0.1 to -0.1) | 1.1 (0.9 to 1.4) | 0.9 (0.7 to 1.1) | -0.5 (-0.6 to -0.3) | 26.7 (21.5 to 33.0) | 26.0 (20.9 to 32.3) | -0.5 (-0.6 to -0.3) |
| Bhutan | 247.9  (202.6 to 301.9) | 33.7  (27.9 to 40.5) | 0.0  (0.0 to 0.0) | 11.2 (6.4 to 20.1) | 2.0 (1.1 to 3.4) | -0.2 (-0.5 to 0.3) | 368.8 (204.6 to 663.0) | 54.4 (30.7 to 97.4) | -0.3 (-0.6 to 0.2) |
| Bolivia (Plurinational State of) | 4626.8  (3857.9 to 5548.1) | 44.6  (37.5 to 52.6) | -0.1  (-0.1 to -0.1) | 355.9 (255.3 to 498.4) | 4.1 (2.9 to 5.7) | -0.3 (-0.5 to 0.0) | 10473.2 (7425.3 to 14501.1) | 105.9 (76.1 to 146.4) | -0.4 (-0.6 to -0.1) |
| Bosnia and Herzegovina | 2059.0  (1721.8 to 2412.8) | 45.0  (37.6 to 52.8) | 0.0  (0.0 to 0.0) | 120.7 (56.0 to 172.9) | 2.1 (1.0 to 3.0) | -0.1 (-0.4 to 0.3) | 2837.4 (1478.0 to 3989.0) | 53.9 (29.1 to 75.4) | -0.1 (-0.4 to 0.2) |
| Botswana | 403.3  (330.7 to 493.6) | 20.2  (16.9 to 24.1) | 0.0  (-0.1 to 0.0) | 21.7 (14.1 to 31.6) | 1.3 (0.9 to 1.9) | -0.1 (-0.5 to 0.5) | 842.1 (535.6 to 1280.5) | 42.3 (28.0 to 61.3) | -0.1 (-0.5 to 0.5) |
| Brazil | 46320.5  (40839.6 to 52387.9) | 19.4  (17.2 to 21.9) | 0.0  (0.0 to 0.0) | 5445.2 (4724.5 to 5900.5) | 2.3 (2.0 to 2.5) | 0.0 (-0.2 to 0.1) | 172622.1 (153155.2 to 185959.0) | 71.1 (63.2 to 76.6) | -0.1 (-0.2 to 0.0) |
| Brunei Darussalam | 96.9  (79.2 to 117.8) | 23.0  (19.2 to 27.3) | -0.1  (-0.1 to -0.1) | 3.1 (2.4 to 3.9) | 1.1 (0.9 to 1.4) | -0.2 (-0.4 to 0.0) | 114.6 (89.4 to 145.0) | 28.6 (22.9 to 35.1) | -0.2 (-0.4 to 0.0) |
| Bulgaria | 4496.0  (3758.1 to 5335.7) | 43.4  (36.6 to 51.0) | 0.0  (0.0 to 0.0) | 274.7 (212.7 to 343.6) | 2.2 (1.7 to 2.7) | 0.3 (-0.1 to 0.7) | 7197.0 (5575.8 to 9147.1) | 66.3 (51.2 to 85.5) | 0.2 (-0.1 to 0.6) |
| Burkina Faso | 3752.3  (3127.0 to 4528.2) | 25.6  (21.8 to 29.8) | 0.1  (0.0 to 0.1) | 509.1 (296.7 to 849.7) | 4.6 (2.7 to 7.6) | 0.3 (-0.3 to 1.0) | 19702.2 (11292.4 to 32930.3) | 146.8 (85.2 to 245.2) | 0.3 (-0.3 to 1.2) |
| Burundi | 1592.5  (1292.7 to 1948.1) | 20.8  (17.3 to 24.7) | 0.0  (0.0 to 0.0) | 96.7 (45.9 to 184.0) | 1.8 (0.9 to 3.5) | 0.0 (-0.4 to 0.8) | 3690.4 (1776.1 to 6909.9) | 54.5 (26.2 to 102.5) | 0.0 (-0.4 to 0.9) |
| Cabo Verde | 128.3  (107.6 to 152.4) | 24.6  (20.8 to 29.0) | 0.0  (0.0 to 0.0) | 12.0 (9.1 to 15.6) | 2.5 (1.9 to 3.3) | 0.2 (-0.2 to 0.7) | 414.7 (302.5 to 560.8) | 82.4 (61.0 to 109.7) | 0.1 (-0.3 to 0.6) |
| Cambodia | 3590.1  (2930.5 to 4364.0) | 24.3  (20.0 to 29.0) | 0.0  (0.0 to 0.0) | 177.6 (119.8 to 299.0) | 1.5 (1.0 to 2.4) | -0.2 (-0.5 to 0.0) | 6153.2 (3962.1 to 10727.1) | 42.8 (28.5 to 72.9) | -0.3 (-0.5 to 0.0) |
| Cameroon | 5125.1  (4262.2 to 6180.3) | 25.8  (22.0 to 30.0) | 0.0  (0.0 to 0.0) | 524.9 (290.7 to 817.2) | 3.5 (2.0 to 5.3) | -0.1 (-0.5 to 0.4) | 20701.1 (11457.5 to 32126.1) | 115.5 (64.5 to 178.5) | 0.0 (-0.5 to 0.5) |
| Canada | 29038.3  (24524.5 to 34152.0) | 56.2  (47.5 to 66.1) | 0.0  (-0.1 to 0.0) | 545.8 (463.3 to 636.2) | 0.8 (0.7 to 0.9) | -0.2 (-0.3 to -0.1) | 12203.6 (10702.0 to 13972.5) | 21.2 (18.6 to 24.3) | -0.2 (-0.2 to -0.1) |
| Central African Republic | 765.9  (630.9 to 932.9) | 21.6  (18.3 to 25.5) | 0.0  (-0.1 to 0.0) | 60.2 (28.1 to 133.6) | 2.3 (1.1 to 4.6) | -0.1 (-0.4 to 0.2) | 2346.8 (1072.4 to 5236.8) | 71.6 (34.0 to 156.9) | -0.1 (-0.5 to 0.2) |
| Chad | 2435.3  (2039.7 to 2940.2) | 25.6  (21.9 to 29.8) | 0.0  (0.0 to 0.0) | 261.9 (171.3 to 381.0) | 3.8 (2.5 to 5.4) | 0.1 (-0.2 to 0.5) | 10255.6 (6815.2 to 14907.1) | 123.4 (80.3 to 180.5) | 0.1 (-0.2 to 0.6) |
| Chile | 7360.5  (6673.1 to 8096.6) | 33.5  (30.4 to 36.8) | 0.0  (0.0 to 0.0) | 388.2 (342.8 to 436.6) | 1.7 (1.5 to 1.9) | -0.3 (-0.4 to -0.2) | 9956.2 (8793.8 to 11282.6) | 43.9 (38.8 to 49.5) | -0.4 (-0.5 to -0.3) |
| China | 493765.4  (416704.6 to 578674.5) | 26.8  (22.8 to 31.3) | -0.3  (-0.3 to -0.3) | 10663.6 (8195.9 to 12809.5) | 0.6 (0.5 to 0.7) | -0.5 (-0.6 to -0.3) | 301309.9 (237109.9 to 363324.1) | 16.1 (12.7 to 19.4) | -0.5 (-0.6 to -0.3) |
| Colombia | 18313.6  (15221.1 to 21775.9) | 35.8  (29.8 to 42.7) | -0.1  (-0.1 to -0.1) | 531.0 (400.3 to 688.3) | 1.0 (0.8 to 1.3) | -0.3 (-0.5 to -0.1) | 14689.9 (11236.9 to 19010.0) | 28.3 (21.7 to 36.8) | -0.3 (-0.5 to -0.1) |
| Comoros | 124.9  (103.4 to 152.5) | 20.6  (17.2 to 24.5) | 0.0  (-0.1 to 0.0) | 7.5 (4.1 to 15.2) | 1.5 (0.8 to 3.0) | -0.2 (-0.5 to 0.5) | 249.3 (132.2 to 489.2) | 42.9 (22.9 to 84.5) | -0.2 (-0.5 to 0.8) |
| Congo | 812.0  (657.9 to 994.0) | 20.3  (16.9 to 24.1) | 0.0  (-0.1 to 0.0) | 40.9 (22.7 to 69.4) | 1.4 (0.8 to 2.3) | -0.3 (-0.5 to 0.0) | 1530.7 (869.9 to 2588.4) | 40.6 (22.8 to 68.4) | -0.4 (-0.6 to 0.0) |
| Cook Islands | 5.5  (4.5 to 6.5) | 26.1  (21.7 to 30.9) | 0.0  (-0.1 to 0.0) | 0.4 (0.3 to 0.5) | 1.7 (1.2 to 2.4) | -0.3 (-0.6 to 0.0) | 11.9 (8.3 to 16.5) | 58.0 (39.3 to 81.6) | -0.3 (-0.6 to 0.1) |
| Costa Rica | 2067.2  (1722.8 to 2459.2) | 40.6  (33.9 to 48.5) | 0.0  (-0.1 to 0.0) | 104.5 (78.4 to 134.8) | 2.0 (1.5 to 2.6) | -0.1 (-0.4 to 0.1) | 2869.1 (2152.2 to 3676.1) | 55.5 (41.7 to 71.2) | -0.2 (-0.4 to 0.1) |
| Croatia | 3012.2  (2763.0 to 3287.3) | 45.6  (41.9 to 49.7) | -0.1  (-0.1 to -0.1) | 143.5 (112.8 to 181.2) | 1.7 (1.4 to 2.2) | -0.3 (-0.5 to -0.2) | 3229.9 (2538.4 to 4097.3) | 46.1 (36.1 to 58.9) | -0.4 (-0.6 to -0.3) |
| Cuba | 4303.2  (3605.1 to 5046.2) | 29.2  (24.3 to 34.6) | 0.0  (0.0 to 0.0) | 210.4 (164.6 to 267.4) | 1.2 (0.9 to 1.5) | 0.3 (-0.1 to 0.7) | 5914.5 (4494.8 to 7544.7) | 37.0 (28.0 to 47.0) | 0.2 (-0.1 to 0.5) |
| Cyprus | 378.6  (330.9 to 430.4) | 21.6  (19.0 to 24.4) | 0.0  (-0.1 to 0.0) | 20.1 (12.2 to 25.1) | 1.2 (0.7 to 1.5) | -0.4 (-0.6 to -0.1) | 381.2 (267.6 to 457.5) | 21.2 (14.9 to 25.3) | -0.4 (-0.6 to -0.1) |
| Czechia | 7695.4  (6819.4 to 8711.8) | 50.4  (44.6 to 57.1) | 0.0  (-0.1 to 0.0) | 393.9 (317.2 to 494.5) | 2.1 (1.6 to 2.6) | -0.4 (-0.5 to -0.2) | 10047.2 (8046.6 to 12431.0) | 60.2 (48.2 to 74.7) | -0.4 (-0.5 to -0.3) |
| Cote d'Ivoire | 4524.2  (3753.5 to 5451.1) | 24.7  (21.1 to 28.9) | 0.0  (0.0 to 0.0) | 442.5 (291.5 to 654.2) | 3.1 (2.1 to 4.6) | 0.0 (-0.4 to 0.5) | 17731.2 (11594.3 to 25825.6) | 103.8 (69.0 to 153.4) | 0.0 (-0.4 to 0.5) |
| Democratic People's Republic of Korea | 12273.7  (10002.8 to 14621.7) | 39.8  (33.0 to 47.3) | 0.0  (0.0 to 0.0) | 283.2 (182.4 to 443.6) | 0.9 (0.6 to 1.4) | -0.2 (-0.4 to 0.2) | 8546.4 (5348.2 to 13634.5) | 27.1 (17.2 to 43.3) | -0.2 (-0.5 to 0.2) |
| Democratic Republic of the Congo | 12235.0  (10021.7 to 14970.3) | 20.9  (17.6 to 24.7) | 0.0  (0.0 to 0.0) | 592.7 (329.9 to 1142.7) | 1.4 (0.7 to 2.8) | -0.2 (-0.5 to 0.1) | 23453.7 (13416.1 to 43825.5) | 43.5 (24.6 to 83.1) | -0.2 (-0.4 to 0.2) |
| Denmark | 1738.3  (1516.8 to 1999.5) | 21.6  (18.5 to 24.9) | 0.0  (0.0 to 0.0) | 158.0 (132.9 to 178.7) | 1.5 (1.2 to 1.7) | -0.1 (-0.2 to 0.1) | 3577.5 (2759.1 to 4075.6) | 38.6 (28.7 to 44.3) | -0.2 (-0.3 to 0.0) |
| Djibouti | 185.5  (148.9 to 228.1) | 19.4  (16.1 to 23.2) | 0.0  (-0.1 to 0.0) | 7.6 (3.8 to 14.4) | 1.1 (0.6 to 2.1) | -0.2 (-0.5 to 0.3) | 288.3 (145.6 to 540.7) | 32.5 (17.0 to 60.8) | -0.2 (-0.5 to 0.3) |
| Dominica | 22.8  (19.0 to 26.8) | 29.0  (24.2 to 34.6) | 0.0  (-0.1 to 0.0) | 1.3 (1.0 to 1.7) | 1.5 (1.1 to 2.0) | -0.2 (-0.4 to 0.1) | 37.0 (27.9 to 49.5) | 46.0 (34.6 to 61.2) | -0.2 (-0.4 to 0.2) |
| Dominican Republic | 2760.9  (2276.2 to 3324.6) | 26.6  (22.0 to 31.7) | 0.0  (0.0 to 0.0) | 79.9 (53.3 to 111.5) | 0.8 (0.6 to 1.2) | -0.1 (-0.4 to 0.4) | 2678.6 (1709.3 to 3782.6) | 25.9 (16.8 to 36.3) | -0.1 (-0.4 to 0.3) |
| Ecuador | 7753.5  (7416.0 to 8092.7) | 47.1  (45.0 to 49.2) | 0.1  (0.0 to 0.1) | 449.2 (326.1 to 699.1) | 3.0 (2.2 to 4.7) | -0.5 (-0.7 to 0.1) | 13308.7 (9710.7 to 20053.0) | 81.7 (59.5 to 123.0) | -0.5 (-0.7 to -0.1) |
| Egypt | 21875.0  (17963.5 to 26390.7) | 27.5  (23.0 to 32.4) | 0.0  (0.0 to 0.0) | 571.5 (307.0 to 877.0) | 1.1 (0.6 to 1.7) | -0.1 (-0.5 to 0.4) | 16098.7 (9069.9 to 23749.7) | 23.7 (13.5 to 35.0) | -0.1 (-0.4 to 0.3) |
| El Salvador | 2262.7  (1894.4 to 2715.3) | 36.9  (30.9 to 44.2) | -0.1  (-0.1 to -0.1) | 93.4 (67.7 to 127.0) | 1.5 (1.1 to 2.1) | -0.4 (-0.6 to -0.1) | 2733.2 (1932.5 to 3843.5) | 45.2 (31.8 to 63.7) | -0.5 (-0.6 to -0.2) |
| Equatorial Guinea | 198.0  (159.9 to 245.2) | 21.2  (17.7 to 25.0) | 0.0  (-0.1 to 0.0) | 7.3 (3.4 to 14.2) | 1.3 (0.6 to 2.4) | -0.4 (-0.6 to -0.1) | 289.0 (131.1 to 548.2) | 36.5 (17.4 to 69.1) | -0.5 (-0.7 to -0.1) |
| Eritrea | 949.3  (766.4 to 1170.9) | 20.5  (17.2 to 24.5) | 0.0  (0.0 to 0.0) | 51.2 (26.8 to 86.7) | 1.6 (0.8 to 2.7) | 0.1 (-0.3 to 0.6) | 2041.2 (1091.6 to 3469.3) | 48.1 (25.7 to 80.1) | 0.1 (-0.3 to 0.7) |
| Estonia | 1177.9  (990.5 to 1386.7) | 62.8  (53.0 to 73.7) | 0.0  (0.0 to 0.0) | 44.2 (32.3 to 57.7) | 2.0 (1.5 to 2.6) | -0.3 (-0.4 to 0.0) | 1295.0 (981.3 to 1663.0) | 69.7 (52.8 to 90.3) | -0.3 (-0.5 to -0.1) |
| Eswatini | 175.2  (142.3 to 216.0) | 20.2  (16.8 to 24.0) | 0.0  (0.0 to 0.0) | 9.3 (5.2 to 14.3) | 1.4 (0.8 to 2.0) | 0.1 (-0.4 to 0.8) | 360.7 (197.2 to 574.6) | 43.7 (24.4 to 67.6) | 0.2 (-0.4 to 0.9) |
| Ethiopia | 15756.1  (12703.5 to 19479.1) | 21.9  (18.2 to 26.2) | 0.0  (0.0 to 0.0) | 625.0 (344.2 to 1232.4) | 1.4 (0.7 to 2.7) | -0.3 (-0.6 to 0.0) | 22235.0 (12442.2 to 41727.6) | 37.5 (21.0 to 73.3) | -0.4 (-0.6 to 0.0) |
| Fiji | 200.8  (163.2 to 242.3) | 23.9  (19.7 to 28.4) | 0.0  (-0.1 to 0.0) | 4.3 (3.2 to 5.9) | 0.6 (0.5 to 0.8) | -0.3 (-0.6 to 0.1) | 155.5 (114.0 to 218.5) | 18.4 (13.6 to 25.3) | -0.3 (-0.6 to 0.0) |
| Finland | 2725.0  (2468.3 to 3028.9) | 33.5  (30.2 to 37.1) | 0.1  (0.0 to 0.1) | 179.0 (155.2 to 201.9) | 1.8 (1.4 to 2.0) | -0.1 (-0.3 to 0.0) | 4712.0 (3566.4 to 5392.4) | 57.7 (38.6 to 66.9) | -0.2 (-0.3 to 0.0) |
| France | 18571.1  (15681.1 to 21737.2) | 19.4  (16.3 to 23.1) | -0.1  (-0.1 to -0.1) | 1186.0 (1023.7 to 1378.0) | 0.9 (0.7 to 1.0) | -0.4 (-0.4 to -0.3) | 23824.5 (20904.0 to 27591.6) | 22.5 (19.7 to 26.1) | -0.4 (-0.4 to -0.3) |
| Gabon | 286.1  (233.0 to 348.5) | 20.0  (16.7 to 24.0) | 0.0  (-0.1 to 0.0) | 13.8 (8.6 to 23.1) | 1.2 (0.8 to 2.0) | -0.3 (-0.5 to 0.0) | 490.4 (306.4 to 808.5) | 36.0 (22.8 to 59.6) | -0.3 (-0.6 to 0.0) |
| Gambia | 386.9  (322.2 to 466.6) | 25.2  (21.5 to 29.4) | 0.0  (0.0 to 0.0) | 36.6 (25.8 to 52.7) | 3.2 (2.3 to 4.6) | -0.1 (-0.5 to 0.5) | 1378.5 (947.8 to 2013.4) | 104.5 (72.7 to 150.1) | -0.1 (-0.5 to 0.6) |
| Georgia | 1287.3  (1171.9 to 1425.3) | 26.8  (24.2 to 29.7) | 0.0  (-0.1 to 0.0) | 43.1 (28.7 to 54.1) | 0.8 (0.5 to 0.9) | 0.8 (0.1 to 1.4) | 1130.3 (808.1 to 1400.2) | 22.7 (16.7 to 28.1) | 1.0 (0.0 to 1.6) |
| Germany | 37430.8  (31649.9 to 43580.5) | 29.1  (24.5 to 33.9) | 0.2  (0.1 to 0.2) | 2173.3 (1890.3 to 2784.8) | 1.2 (1.1 to 1.5) | -0.3 (-0.4 to -0.2) | 47104.4 (41502.4 to 56466.2) | 32.6 (28.8 to 37.7) | -0.3 (-0.4 to -0.2) |
| Ghana | 6041.9  (5022.5 to 7223.2) | 25.0  (21.2 to 29.2) | 0.0  (0.0 to 0.0) | 754.2 (453.4 to 1109.1) | 4.0 (2.4 to 5.7) | 0.0 (-0.5 to 0.5) | 28361.4 (17357.9 to 42418.0) | 126.5 (77.2 to 186.5) | 0.0 (-0.4 to 0.6) |
| Greece | 3482.9  (2941.7 to 4054.1) | 21.0  (17.5 to 24.7) | 0.0  (-0.1 to 0.0) | 349.4 (270.1 to 407.1) | 1.3 (1.1 to 1.4) | -0.1 (-0.2 to 0.0) | 5577.3 (4778.6 to 6288.1) | 26.6 (23.5 to 30.0) | -0.1 (-0.2 to 0.0) |
| Greenland | 36.2  (30.0 to 43.2) | 55.5  (46.6 to 65.0) | 0.0  (-0.1 to 0.0) | 0.9 (0.7 to 1.2) | 1.3 (1.0 to 1.7) | -0.4 (-0.6 to 0.0) | 29.7 (22.8 to 39.6) | 42.1 (32.4 to 56.7) | -0.4 (-0.6 to 0.0) |
| Grenada | 29.0  (24.1 to 34.6) | 26.3  (22.0 to 31.4) | -0.1  (-0.1 to -0.1) | 1.0 (0.8 to 1.2) | 0.9 (0.8 to 1.1) | -0.1 (-0.3 to 0.1) | 31.8 (26.6 to 37.5) | 27.9 (23.5 to 32.8) | -0.2 (-0.3 to 0.1) |
| Guam | 44.5  (36.7 to 53.2) | 24.9  (20.5 to 29.8) | 0.0  (-0.1 to 0.0) | 1.0 (0.7 to 1.4) | 0.6 (0.4 to 0.8) | -0.2 (-0.5 to 0.0) | 34.3 (22.7 to 45.0) | 19.3 (12.8 to 25.3) | 0.0 (-0.3 to 0.5) |
| Guatemala | 5915.5  (4929.7 to 7094.2) | 39.7  (33.5 to 47.0) | -0.1  (-0.1 to -0.1) | 395.9 (304.5 to 512.2) | 3.0 (2.3 to 3.8) | -0.4 (-0.5 to -0.1) | 16522.2 (12650.4 to 21338.9) | 108.4 (83.1 to 139.8) | -0.4 (-0.6 to -0.1) |
| Guinea | 1974.1  (1633.7 to 2391.3) | 23.7  (20.0 to 27.8) | 0.0  (0.0 to 0.0) | 182.6 (122.1 to 265.0) | 2.8 (1.9 to 4.1) | 0.0 (-0.3 to 0.5) | 6950.9 (4623.1 to 10031.6) | 93.4 (62.9 to 136.7) | 0.0 (-0.3 to 0.6) |
| Guinea-Bissau | 321.1  (266.8 to 387.2) | 25.4  (21.8 to 29.7) | 0.0  (-0.1 to 0.0) | 44.5 (28.7 to 64.7) | 4.8 (3.2 to 6.8) | -0.1 (-0.4 to 0.5) | 1756.7 (1116.0 to 2561.8) | 160.0 (103.5 to 233.2) | -0.1 (-0.4 to 0.5) |
| Guyana | 215.2  (179.2 to 258.5) | 29.9  (25.1 to 35.5) | 0.0  (0.0 to 0.0) | 15.8 (11.7 to 20.2) | 2.4 (1.8 to 3.0) | 0.0 (-0.3 to 0.3) | 584.2 (435.6 to 753.0) | 79.4 (59.3 to 102.0) | 0.0 (-0.3 to 0.4) |
| Haiti | 2925.0  (2403.0 to 3525.9) | 29.3  (24.4 to 34.7) | 0.0  (0.0 to 0.0) | 156.7 (102.4 to 242.4) | 2.0 (1.3 to 3.1) | -0.2 (-0.5 to 0.1) | 5881.5 (3857.0 to 8889.7) | 61.4 (40.4 to 93.4) | -0.3 (-0.5 to 0.0) |
| Honduras | 3274.1  (2728.0 to 3959.1) | 40.2  (33.9 to 47.7) | 0.0  (0.0 to 0.0) | 253.3 (179.2 to 353.5) | 4.1 (2.9 to 5.5) | 0.1 (-0.2 to 0.6) | 7963.6 (5387.7 to 11645.7) | 109.2 (75.2 to 156.0) | -0.1 (-0.4 to 0.2) |
| Hungary | 6337.4  (5319.7 to 7449.6) | 45.0  (37.9 to 52.7) | -0.1  (-0.1 to -0.1) | 378.5 (306.8 to 470.7) | 2.2 (1.8 to 2.7) | -0.5 (-0.6 to -0.3) | 10049.4 (8095.8 to 12472.4) | 66.7 (53.9 to 83.7) | -0.6 (-0.6 to -0.4) |
| Iceland | 98.4  (83.4 to 115.8) | 22.3  (18.8 to 26.3) | 0.1  (0.1 to 0.1) | 3.6 (3.0 to 4.3) | 0.7 (0.6 to 0.8) | -0.4 (-0.5 to -0.3) | 86.1 (74.3 to 101.2) | 18.5 (15.9 to 21.7) | -0.4 (-0.5 to -0.3) |
| India | 618862.3  (508186.9 to 749921.1) | 45.2  (37.7 to 54.4) | 0.2  (0.1 to 0.2) | 20455.9 (15269.6 to 25749.7) | 1.8 (1.3 to 2.2) | -0.2 (-0.4 to 0.2) | 715968.6 (539262.1 to 897634.1) | 54.5 (41.0 to 68.2) | -0.2 (-0.4 to 0.1) |
| Indonesia | 72002.1  (58941.4 to 86950.0) | 27.8  (23.1 to 33.1) | 0.0  (0.0 to 0.0) | 3439.9 (2628.5 to 5523.3) | 1.8 (1.4 to 2.7) | -0.2 (-0.4 to 0.1) | 106212.3 (78050.3 to 177934.2) | 44.4 (33.3 to 71.6) | -0.3 (-0.5 to -0.1) |
| Iran (Islamic Republic of) | 19554.6  (16084.2 to 23288.8) | 23.9  (20.0 to 28.2) | 0.0  (0.0 to 0.0) | 398.3 (308.3 to 479.8) | 0.6 (0.5 to 0.7) | -0.3 (-0.5 to 0.1) | 10144.4 (8531.4 to 12698.3) | 13.2 (10.9 to 16.2) | -0.3 (-0.4 to 0.0) |
| Iraq | 7590.9  (6223.0 to 9256.6) | 23.8  (19.7 to 28.2) | -0.1  (-0.1 to -0.1) | 98.7 (72.7 to 136.3) | 0.4 (0.3 to 0.6) | -0.1 (-0.4 to 0.3) | 3519.4 (2578.7 to 4716.8) | 11.8 (8.9 to 15.6) | -0.2 (-0.5 to 0.3) |
| Ireland | 1160.2  (961.7 to 1366.9) | 18.9  (15.7 to 22.3) | 0.0  (0.0 to 0.0) | 48.2 (40.7 to 54.7) | 0.7 (0.6 to 0.7) | -0.1 (-0.3 to 0.0) | 1148.2 (964.1 to 1316.4) | 17.2 (14.4 to 19.8) | -0.2 (-0.3 to 0.0) |
| Israel | 2003.2  (1671.8 to 2353.6) | 19.8  (16.4 to 23.3) | 0.0  (0.0 to 0.0) | 109.4 (87.3 to 127.6) | 0.9 (0.7 to 1.0) | -0.1 (-0.2 to 0.2) | 1974.2 (1730.1 to 2220.0) | 17.7 (15.6 to 19.9) | -0.1 (-0.2 to 0.0) |
| Italy | 27615.2  (24442.5 to 31441.9) | 25.6  (22.9 to 28.7) | -0.1  (-0.1 to -0.1) | 1304.9 (1137.5 to 1480.7) | 0.8 (0.7 to 1.0) | -0.3 (-0.4 to -0.2) | 22720.3 (20324.9 to 27552.7) | 18.8 (16.9 to 24.4) | -0.4 (-0.4 to -0.3) |
| Jamaica | 781.2  (645.6 to 940.5) | 26.1  (21.6 to 31.4) | 0.0  (-0.1 to 0.0) | 17.6 (13.5 to 22.2) | 0.6 (0.4 to 0.7) | 0.0 (-0.3 to 0.3) | 543.6 (415.3 to 695.8) | 18.1 (13.8 to 23.2) | 0.0 (-0.3 to 0.3) |
| Japan | 60692.6  (53258.4 to 68866.5) | 36.2  (31.6 to 41.8) | 0.0  (0.0 to 0.0) | 1655.1 (1317.6 to 2231.5) | 0.4 (0.4 to 0.6) | -0.5 (-0.5 to -0.3) | 33114.0 (28256.9 to 41215.3) | 14.0 (11.9 to 17.2) | -0.4 (-0.5 to -0.3) |
| Jordan | 3806.6  (3194.9 to 4542.7) | 40.4  (34.3 to 47.3) | 0.1  (0.0 to 0.1) | 47.4 (36.6 to 59.0) | 0.9 (0.7 to 1.1) | -0.5 (-0.7 to -0.3) | 1456.1 (1129.2 to 1797.1) | 19.3 (15.0 to 23.5) | -0.5 (-0.6 to -0.3) |
| Kazakhstan | 8054.0  (6859.1 to 9290.2) | 42.9  (36.9 to 49.4) | 0.0  (-0.1 to 0.0) | 881.1 (678.9 to 1073.7) | 5.0 (3.8 to 6.0) | -0.2 (-0.4 to 0.0) | 31211.9 (24591.8 to 38898.6) | 161.9 (126.9 to 200.1) | -0.2 (-0.3 to 0.0) |
| Kenya | 8680.4  (7041.3 to 10651.2) | 23.9  (20.0 to 28.5) | 0.0  (0.0 to 0.0) | 432.4 (231.8 to 701.5) | 1.8 (0.9 to 2.9) | 0.0 (-0.3 to 0.5) | 15564.0 (8640.7 to 24586.9) | 50.2 (27.3 to 80.6) | 0.0 (-0.3 to 0.5) |
| Kiribati | 25.4  (21.0 to 30.8) | 28.0  (23.5 to 32.8) | 0.0  (0.0 to 0.0) | 2.3 (1.5 to 3.5) | 2.9 (2.0 to 4.5) | -0.2 (-0.4 to 0.1) | 99.7 (60.9 to 146.0) | 100.0 (64.9 to 146.5) | -0.2 (-0.4 to 0.1) |
| Kuwait | 1205.7  (949.4 to 1474.8) | 28.2  (23.4 to 33.3) | 0.0  (0.0 to 0.0) | 22.4 (17.7 to 27.9) | 0.8 (0.6 to 1.0) | -0.1 (-0.4 to 0.1) | 794.6 (628.2 to 981.0) | 21.2 (16.9 to 26.1) | -0.2 (-0.4 to 0.0) |
| Kyrgyzstan | 1835.3  (1506.4 to 2184.9) | 32.2  (26.6 to 38.1) | 0.0  (0.0 to 0.0) | 85.8 (67.9 to 103.3) | 1.6 (1.3 to 1.9) | 0.2 (-0.1 to 0.5) | 3473.2 (2687.9 to 4252.7) | 58.5 (46.1 to 70.7) | 0.2 (-0.1 to 0.5) |
| Lao People's Democratic Republic | 1465.3  (1197.3 to 1775.5) | 24.2  (20.1 to 28.7) | 0.0  (0.0 to 0.0) | 67.8 (43.1 to 104.5) | 1.4 (0.9 to 2.1) | -0.3 (-0.5 to 0.2) | 2534.8 (1597.7 to 4011.9) | 43.1 (27.4 to 67.3) | -0.3 (-0.6 to 0.1) |
| Latvia | 1655.3  (1472.1 to 1848.1) | 61.7  (54.6 to 69.4) | 0.0  (-0.1 to 0.0) | 100.2 (80.7 to 125.7) | 3.3 (2.6 to 4.2) | 0.1 (-0.2 to 0.4) | 3219.1 (2542.3 to 4053.2) | 126.8 (99.5 to 161.3) | 0.1 (-0.1 to 0.4) |
| Lebanon | 1423.3  (1173.9 to 1688.8) | 26.9  (22.3 to 31.9) | 0.0  (-0.1 to 0.0) | 36.4 (21.4 to 52.0) | 0.7 (0.4 to 1.0) | -0.2 (-0.5 to 0.1) | 804.6 (506.4 to 1119.2) | 15.3 (9.6 to 21.3) | -0.3 (-0.5 to 0.1) |
| Lesotho | 363.6  (299.0 to 443.7) | 21.1  (17.7 to 25.2) | 0.0  (0.0 to 0.0) | 23.2 (15.5 to 33.9) | 1.7 (1.1 to 2.3) | 0.2 (-0.3 to 1.0) | 863.3 (552.6 to 1307.7) | 52.4 (34.4 to 77.7) | 0.3 (-0.3 to 1.2) |
| Liberia | 812.4  (670.2 to 986.1) | 23.5  (19.9 to 27.7) | 0.0  (0.0 to 0.0) | 83.6 (51.7 to 123.3) | 3.2 (2.1 to 4.4) | 0.0 (-0.4 to 0.5) | 3219.4 (1981.4 to 4839.7) | 98.7 (61.7 to 143.7) | 0.0 (-0.4 to 0.5) |
| Libya | 1770.6  (1428.1 to 2129.5) | 27.4  (22.7 to 32.5) | 0.0  (0.0 to 0.0) | 38.9 (24.5 to 55.5) | 0.8 (0.5 to 1.2) | -0.2 (-0.5 to 0.2) | 1059.5 (686.1 to 1486.6) | 18.3 (12.0 to 25.4) | -0.2 (-0.5 to 0.2) |
| Lithuania | 2647.7  (2371.9 to 2950.2) | 64.8  (57.8 to 72.5) | 0.1  (0.1 to 0.1) | 183.5 (131.7 to 231.2) | 4.0 (2.9 to 5.1) | 0.6 (0.0 to 1.1) | 5429.8 (3960.5 to 6988.2) | 141.2 (105.4 to 182.2) | 0.6 (0.0 to 1.1) |
| Luxembourg | 242.0  (217.8 to 268.1) | 29.2  (26.2 to 32.3) | 0.1  (0.0 to 0.1) | 7.8 (6.5 to 9.7) | 0.8 (0.6 to 1.0) | -0.4 (-0.5 to -0.3) | 183.6 (152.1 to 226.1) | 20.5 (17.0 to 25.2) | -0.4 (-0.5 to -0.3) |
| Madagascar | 3564.5  (2887.1 to 4384.8) | 19.5  (16.2 to 23.4) | 0.0  (0.0 to 0.0) | 148.1 (84.1 to 264.5) | 1.2 (0.7 to 2.2) | 0.1 (-0.4 to 0.7) | 5775.9 (3393.0 to 9943.9) | 34.8 (20.3 to 61.5) | 0.0 (-0.4 to 0.7) |
| Malawi | 2494.2  (2021.5 to 3084.4) | 20.6  (17.2 to 24.5) | 0.0  (0.0 to 0.0) | 130.4 (74.6 to 236.9) | 1.5 (0.9 to 2.8) | 0.0 (-0.4 to 0.6) | 4930.7 (2846.8 to 8870.4) | 46.2 (26.7 to 84.1) | 0.0 (-0.4 to 0.6) |
| Malaysia | 8262.1  (6863.2 to 9875.6) | 26.8  (22.4 to 31.6) | 0.0  (0.0 to 0.0) | 635.2 (469.0 to 838.1) | 2.5 (1.8 to 3.3) | 0.0 (-0.3 to 0.4) | 18361.6 (13311.6 to 25261.7) | 62.7 (45.8 to 83.6) | -0.1 (-0.3 to 0.3) |
| Maldives | 103.9  (81.4 to 128.7) | 20.2  (16.5 to 24.3) | 0.0  (-0.1 to 0.0) | 1.6 (1.2 to 2.2) | 0.5 (0.3 to 0.6) | -0.5 (-0.7 to -0.2) | 60.3 (44.6 to 88.3) | 12.8 (9.9 to 17.0) | -0.6 (-0.7 to -0.3) |
| Mali | 3446.3  (2851.6 to 4144.4) | 25.3  (21.6 to 29.6) | 0.1  (0.0 to 0.1) | 355.8 (229.4 to 566.6) | 3.5 (2.3 to 5.5) | 0.1 (-0.4 to 0.9) | 13776.9 (8759.1 to 22074.3) | 113.3 (72.5 to 181.8) | 0.1 (-0.4 to 0.9) |
| Malta | 123.1  (108.3 to 141.5) | 18.0  (15.7 to 20.4) | 0.0  (0.0 to 0.0) | 6.7 (5.6 to 7.9) | 0.8 (0.6 to 0.9) | -0.3 (-0.4 to -0.2) | 145.5 (123.4 to 171.4) | 20.0 (17.1 to 23.6) | -0.3 (-0.4 to -0.1) |
| Marshall Islands | 12.2  (9.9 to 15.0) | 26.6  (22.1 to 31.5) | 0.0  (-0.1 to 0.0) | 0.7 (0.4 to 1.1) | 1.7 (1.1 to 2.8) | -0.2 (-0.5 to 0.1) | 27.2 (16.1 to 45.8) | 55.9 (34.7 to 92.3) | -0.2 (-0.5 to 0.1) |
| Mauritania | 702.4  (585.9 to 847.0) | 24.0  (20.2 to 28.3) | 0.0  (0.0 to 0.0) | 55.9 (37.0 to 84.8) | 2.5 (1.7 to 3.6) | -0.4 (-0.6 to -0.1) | 1951.4 (1214.3 to 3133.6) | 75.3 (48.6 to 116.3) | -0.4 (-0.6 to -0.1) |
| Mauritius | 377.1  (313.9 to 448.6) | 24.4  (20.3 to 29.1) | -0.1  (-0.1 to -0.1) | 31.0 (23.3 to 40.5) | 1.9 (1.5 to 2.5) | -0.4 (-0.6 to -0.2) | 1203.1 (906.7 to 1558.3) | 77.6 (58.6 to 99.8) | -0.4 (-0.6 to -0.2) |
| Mexico | 50891.2  (44796.3 to 57815.6) | 40.4  (35.6 to 45.6) | 0.1  (0.1 to 0.1) | 2511.3 (2125.7 to 2976.6) | 2.1 (1.8 to 2.5) | 0.0 (-0.2 to 0.1) | 82889.2 (70474.1 to 98314.7) | 65.7 (55.9 to 77.8) | -0.1 (-0.2 to 0.1) |
| Micronesia (Federated States of) | 22.4  (18.5 to 27.0) | 26.5  (22.2 to 31.4) | 0.0  (-0.1 to 0.0) | 1.3 (0.7 to 2.2) | 1.8 (1.1 to 2.9) | -0.3 (-0.6 to 0.2) | 50.2 (26.7 to 89.8) | 56.6 (31.7 to 98.2) | -0.3 (-0.6 to 0.2) |
| Monaco | 11.9  (9.8 to 14.1) | 19.5  (16.3 to 23.2) | 0.0  (0.0 to 0.0) | 0.6 (0.5 to 0.9) | 0.7 (0.5 to 0.9) | -0.1 (-0.4 to 0.2) | 11.8 (8.6 to 15.1) | 16.2 (11.9 to 20.9) | -0.1 (-0.4 to 0.3) |
| Mongolia | 1194.1  (990.7 to 1413.5) | 38.6  (32.9 to 44.8) | 0.0  (-0.1 to 0.0) | 104.5 (55.2 to 149.4) | 4.5 (2.1 to 6.3) | -0.2 (-0.5 to 0.2) | 3676.1 (2081.0 to 5204.2) | 120.6 (66.3 to 170.1) | -0.2 (-0.5 to 0.2) |
| Montenegro | 336.4  (280.9 to 395.6) | 42.4  (35.3 to 50.0) | 0.0  (-0.1 to 0.0) | 13.7 (11.0 to 17.5) | 1.5 (1.2 to 2.0) | -0.1 (-0.3 to 0.2) | 420.9 (339.3 to 527.4) | 50.8 (41.0 to 63.8) | -0.1 (-0.3 to 0.1) |
| Morocco | 9292.0  (7656.8 to 11079.5) | 27.1  (22.5 to 32.0) | 0.0  (0.0 to 0.0) | 251.2 (171.6 to 343.7) | 1.0 (0.6 to 1.3) | -0.1 (-0.4 to 0.3) | 6243.2 (4598.2 to 8592.1) | 20.0 (14.7 to 27.0) | -0.2 (-0.4 to 0.2) |
| Mozambique | 3888.7  (3171.7 to 4795.3) | 21.1  (17.7 to 25.1) | 0.0  (0.0 to 0.0) | 274.1 (157.3 to 514.7) | 2.1 (1.2 to 3.9) | 0.3 (-0.2 to 1.1) | 10628.9 (6233.6 to 19861.3) | 64.9 (37.5 to 121.0) | 0.4 (-0.2 to 1.2) |
| Myanmar | 11496.9  (9408.2 to 13908.2) | 21.6  (17.9 to 26.0) | 0.0  (-0.1 to 0.0) | 364.3 (232.2 to 672.5) | 0.7 (0.5 to 1.3) | -0.3 (-0.6 to 0.0) | 13962.1 (8537.1 to 27635.7) | 25.7 (16.0 to 49.7) | -0.4 (-0.6 to -0.1) |
| Namibia | 405.5  (334.5 to 492.5) | 21.1  (17.6 to 25.1) | 0.0  (0.0 to 0.0) | 20.5 (13.3 to 30.8) | 1.3 (0.9 to 1.9) | -0.1 (-0.4 to 0.3) | 723.2 (449.5 to 1147.2) | 40.1 (25.7 to 61.6) | -0.1 (-0.4 to 0.4) |
| Nauru | 1.9  (1.5 to 2.3) | 25.8  (21.5 to 30.5) | 0.0  (0.0 to 0.0) | 0.1 (0.1 to 0.2) | 1.8 (1.1 to 2.9) | -0.2 (-0.4 to 0.2) | 4.7 (2.5 to 8.7) | 59.5 (33.5 to 103.2) | -0.1 (-0.4 to 0.2) |
| Nepal | 6949.1  (6074.0 to 7974.9) | 25.9  (22.7 to 29.4) | 0.0  (-0.1 to 0.0) | 531.3 (346.7 to 739.4) | 2.5 (1.6 to 3.5) | -0.3 (-0.5 to 0.2) | 16402.4 (10667.4 to 22800.3) | 65.1 (42.3 to 90.2) | -0.4 (-0.6 to 0.0) |
| Netherlands | 2259.0  (1881.9 to 2668.5) | 8.8  (7.2 to 10.3) | 0.0  (-0.1 to 0.0) | 269.1 (235.3 to 315.1) | 0.8 (0.7 to 0.9) | -0.2 (-0.3 to -0.1) | 5412.1 (4794.8 to 6186.7) | 18.5 (16.4 to 21.0) | -0.2 (-0.3 to -0.1) |
| New Zealand | 2243.6  (2009.1 to 2517.0) | 37.9  (33.9 to 42.4) | 0.1  (0.1 to 0.1) | 59.0 (49.3 to 67.7) | 0.7 (0.6 to 0.8) | -0.2 (-0.3 to -0.1) | 1234.1 (1054.4 to 1400.4) | 17.9 (15.4 to 20.3) | -0.3 (-0.3 to -0.2) |
| Nicaragua | 2089.0  (1710.2 to 2528.8) | 36.0  (29.8 to 43.1) | -0.1  (-0.1 to -0.1) | 67.6 (47.0 to 88.6) | 1.5 (1.1 to 2.0) | 0.0 (-0.3 to 0.5) | 2089.1 (1446.8 to 2764.2) | 40.2 (28.2 to 52.5) | -0.2 (-0.4 to 0.3) |
| Niger | 3441.5  (2859.0 to 4169.3) | 26.2  (22.5 to 30.4) | 0.0  (0.0 to 0.0) | 361.3 (216.2 to 572.3) | 3.8 (2.3 to 6.0) | 0.1 (-0.2 to 0.6) | 14242.0 (8555.0 to 22191.8) | 124.2 (74.2 to 198.3) | 0.1 (-0.3 to 0.6) |
| Nigeria | 41313.2  (34126.9 to 49830.5) | 28.5  (24.3 to 33.6) | 0.0  (0.0 to 0.0) | 3882.1 (2087.2 to 7494.1) | 3.7 (2.1 to 6.9) | -0.1 (-0.4 to 0.3) | 148822.2 (79262.8 to 285452.4) | 114.2 (61.8 to 220.5) | -0.2 (-0.4 to 0.2) |
| Niue | 0.5  (0.4 to 0.6) | 26.1  (21.7 to 31.0) | 0.0  (0.0 to 0.0) | 0.0 (0.0 to 0.0) | 1.3 (0.9 to 1.7) | -0.2 (-0.5 to 0.2) | 0.7 (0.5 to 1.0) | 38.7 (25.7 to 55.4) | -0.2 (-0.5 to 0.2) |
| North Macedonia | 1162.0  (972.4 to 1376.1) | 42.3  (35.8 to 49.9) | 0.0  (-0.1 to 0.0) | 49.4 (35.6 to 64.2) | 1.7 (1.2 to 2.2) | 0.0 (-0.3 to 0.4) | 1345.7 (962.1 to 1746.1) | 45.9 (32.7 to 59.4) | -0.1 (-0.3 to 0.2) |
| Northern Mariana Islands | 12.8  (10.4 to 15.5) | 26.5  (22.0 to 31.5) | 0.0  (0.0 to 0.0) | 0.6 (0.4 to 0.8) | 1.3 (0.8 to 1.6) | 0.2 (-0.5 to 1.2) | 19.7 (12.0 to 26.9) | 38.3 (23.5 to 52.4) | 0.4 (-0.5 to 1.8) |
| Norway | 2857.2  (2480.3 to 3283.7) | 40.3  (35.0 to 46.5) | 0.3  (0.3 to 0.3) | 69.7 (56.6 to 79.9) | 0.7 (0.6 to 0.8) | -0.3 (-0.3 to -0.2) | 1550.8 (1321.0 to 1777.3) | 18.5 (15.9 to 21.4) | -0.3 (-0.3 to -0.2) |
| Oman | 930.8  (722.8 to 1168.8) | 24.7  (20.4 to 29.3) | 0.0  (-0.1 to 0.0) | 5.9 (3.4 to 8.4) | 0.4 (0.3 to 0.6) | -0.3 (-0.6 to 0.2) | 228.9 (147.5 to 335.6) | 9.3 (5.8 to 12.2) | -0.3 (-0.6 to 0.0) |
| Pakistan | 68167.5  (55967.5 to 82920.8) | 39.0  (32.7 to 46.7) | 0.1  (0.0 to 0.1) | 2479.0 (1542.0 to 3465.0) | 2.1 (1.3 to 2.9) | -0.1 (-0.4 to 0.4) | 94460.1 (58985.7 to 133123.9) | 60.8 (38.4 to 84.0) | -0.1 (-0.4 to 0.4) |
| Palau | 5.5  (4.4 to 6.7) | 26.4  (21.8 to 31.4) | 0.0  (0.0 to 0.0) | 0.3 (0.1 to 0.4) | 1.3 (0.8 to 1.9) | -0.1 (-0.4 to 0.4) | 9.1 (5.3 to 14.1) | 42.3 (24.7 to 65.2) | 0.0 (-0.4 to 0.5) |
| Palestine | 983.3  (804.5 to 1187.4) | 28.2  (23.5 to 33.3) | 0.0  (-0.1 to 0.0) | 22.9 (16.8 to 28.7) | 1.3 (0.9 to 1.6) | -0.2 (-0.5 to 0.1) | 604.3 (472.1 to 743.9) | 23.4 (17.9 to 29.0) | -0.3 (-0.5 to 0.0) |
| Panama | 1469.7  (1211.5 to 1757.1) | 35.0  (28.9 to 41.9) | 0.0  (-0.1 to 0.0) | 35.3 (25.5 to 46.3) | 0.8 (0.6 to 1.1) | -0.1 (-0.3 to 0.2) | 1067.1 (793.3 to 1405.0) | 25.5 (19.0 to 33.5) | -0.1 (-0.3 to 0.2) |
| Papua New Guinea | 1680.2  (1355.7 to 2061.0) | 23.7  (19.6 to 28.2) | 0.0  (-0.1 to 0.0) | 49.6 (29.7 to 75.5) | 0.8 (0.5 to 1.3) | 0.0 (-0.3 to 0.3) | 2133.8 (1284.8 to 3242.2) | 28.0 (17.2 to 42.1) | 0.0 (-0.3 to 0.4) |
| Paraguay | 1188.6  (990.8 to 1408.0) | 18.7  (15.8 to 22.0) | 0.1  (0.0 to 0.1) | 112.2 (54.1 to 162.8) | 2.0 (0.9 to 2.9) | 0.2 (-0.3 to 0.7) | 3238.1 (1812.4 to 4634.0) | 53.1 (28.5 to 76.2) | 0.1 (-0.3 to 0.6) |
| Peru | 14065.8  (11726.2 to 16648.1) | 41.5  (34.8 to 48.8) | -0.1  (-0.1 to -0.1) | 639.9 (451.3 to 870.3) | 1.9 (1.4 to 2.7) | -0.5 (-0.6 to -0.2) | 19872.9 (13849.9 to 27079.3) | 58.8 (41.0 to 80.3) | -0.5 (-0.7 to -0.3) |
| Philippines | 19310.1  (15600.2 to 23699.2) | 19.0  (15.6 to 23.1) | -0.1  (-0.1 to -0.1) | 766.0 (586.8 to 1022.4) | 0.9 (0.7 to 1.2) | -0.2 (-0.4 to 0.1) | 28716.8 (22083.0 to 40177.1) | 29.5 (22.6 to 40.1) | -0.2 (-0.4 to 0.1) |
| Poland | 21951.2  (19659.7 to 24570.8) | 40.9  (36.5 to 45.7) | -0.2  (-0.2 to -0.2) | 2002.7 (1666.4 to 2363.0) | 3.2 (2.7 to 3.8) | 0.0 (-0.2 to 0.2) | 56016.2 (46056.1 to 67042.9) | 103.0 (84.6 to 123.7) | 0.0 (-0.2 to 0.2) |
| Portugal | 2719.8  (2360.1 to 3121.1) | 15.6  (13.3 to 18.0) | 0.0  (0.0 to 0.0) | 353.7 (290.1 to 401.7) | 1.4 (1.2 to 1.6) | -0.3 (-0.4 to -0.2) | 6287.0 (5399.8 to 7208.2) | 31.5 (27.4 to 36.8) | -0.4 (-0.5 to -0.3) |
| Puerto Rico | 1489.2  (1255.9 to 1738.4) | 30.4  (25.3 to 36.1) | -0.1  (-0.1 to -0.1) | 102.4 (77.4 to 132.1) | 1.5 (1.1 to 1.9) | -0.4 (-0.6 to -0.2) | 2200.1 (1669.4 to 2835.3) | 40.1 (30.1 to 51.7) | -0.4 (-0.5 to -0.2) |
| Qatar | 733.8  (570.1 to 927.5) | 28.7  (24.0 to 34.0) | 0.0  (-0.1 to 0.0) | 9.7 (4.7 to 14.8) | 1.3 (0.8 to 1.8) | -0.2 (-0.5 to 0.1) | 417.2 (214.3 to 625.3) | 25.5 (15.3 to 35.8) | -0.3 (-0.5 to 0.0) |
| Republic of Korea | 16904.3  (14038.6 to 19885.1) | 24.0  (20.1 to 28.6) | -0.1  (-0.1 to -0.1) | 562.1 (414.8 to 693.8) | 0.7 (0.5 to 0.8) | -0.6 (-0.8 to -0.3) | 14067.9 (10802.2 to 18126.8) | 17.7 (13.8 to 22.7) | -0.6 (-0.8 to -0.3) |
| Republic of Moldova | 3465.3  (2942.8 to 4025.2) | 71.3  (60.8 to 82.7) | -0.1  (-0.1 to -0.1) | 230.2 (190.7 to 289.6) | 4.4 (3.7 to 5.5) | -0.4 (-0.5 to -0.3) | 8519.3 (7097.6 to 10265.4) | 173.1 (144.1 to 208.2) | -0.4 (-0.6 to -0.3) |
| Romania | 14037.2  (12346.4 to 15927.2) | 50.8  (44.7 to 57.1) | 0.1  (0.0 to 0.1) | 1042.9 (837.0 to 1308.9) | 3.3 (2.7 to 4.1) | -0.1 (-0.3 to 0.1) | 31145.4 (25299.1 to 38636.7) | 113.1 (91.4 to 139.4) | -0.2 (-0.4 to 0.0) |
| Russian Federation | 157598.7  (133616.2 to 183370.9) | 82.0  (70.1 to 95.1) | 0.1  (0.1 to 0.1) | 11615.3 (9804.0 to 13618.5) | 5.7 (4.8 to 6.7) | 1.1 (0.2 to 1.5) | 409216.7 (343872.3 to 483896.1) | 217.3 (182.5 to 256.5) | 1.1 (0.3 to 1.6) |
| Rwanda | 1965.6  (1589.6 to 2414.4) | 21.4  (17.8 to 25.3) | 0.0  (0.0 to 0.0) | 102.5 (57.6 to 172.8) | 1.5 (0.9 to 2.6) | -0.4 (-0.6 to 0.1) | 3844.0 (2142.1 to 6385.4) | 45.1 (25.6 to 75.1) | -0.4 (-0.6 to 0.1) |
| Saint Kitts and Nevis | 19.9  (16.6 to 23.5) | 30.0  (25.2 to 35.1) | 0.0  (0.0 to 0.0) | 1.8 (1.3 to 2.4) | 2.7 (1.9 to 3.5) | -0.3 (-0.5 to 0.0) | 58.0 (37.5 to 79.2) | 79.9 (51.7 to 108.9) | -0.3 (-0.5 to 0.0) |
| Saint Lucia | 49.9  (41.1 to 59.7) | 24.7  (20.3 to 29.8) | 0.0  (-0.1 to 0.0) | 1.0 (0.8 to 1.3) | 0.5 (0.4 to 0.6) | -0.2 (-0.4 to 0.0) | 34.7 (27.9 to 43.0) | 16.5 (13.2 to 20.3) | -0.1 (-0.3 to 0.1) |
| Saint Vincent and the Grenadines | 35.7  (29.8 to 42.3) | 28.6  (23.8 to 34.0) | 0.0  (-0.1 to 0.0) | 1.9 (1.5 to 2.3) | 1.4 (1.2 to 1.7) | 0.0 (-0.2 to 0.2) | 60.0 (48.6 to 73.0) | 46.1 (37.4 to 56.0) | -0.1 (-0.3 to 0.2) |
| Samoa | 45.1  (37.6 to 54.4) | 26.4  (22.0 to 31.5) | 0.0  (0.0 to 0.0) | 2.0 (1.4 to 2.7) | 1.3 (1.0 to 1.8) | -0.2 (-0.4 to 0.2) | 69.0 (48.3 to 97.6) | 40.3 (28.5 to 56.9) | -0.1 (-0.4 to 0.3) |
| San Marino | 9.8  (8.2 to 11.4) | 20.7  (17.3 to 24.5) | 0.0  (-0.1 to 0.0) | 0.9 (0.6 to 1.2) | 1.1 (0.7 to 1.6) | -0.1 (-0.4 to 0.3) | 12.7 (8.2 to 18.5) | 20.5 (13.3 to 30.4) | -0.1 (-0.4 to 0.4) |
| Sao Tome and Principe | 37.7  (31.2 to 45.4) | 23.7  (20.0 to 28.0) | 0.0  (-0.1 to 0.0) | 2.9 (1.9 to 5.0) | 2.4 (1.5 to 3.9) | -0.2 (-0.5 to 0.4) | 109.1 (68.7 to 191.7) | 73.5 (48.0 to 125.5) | -0.2 (-0.5 to 0.4) |
| Saudi Arabia | 8606.5  (6886.2 to 10574.7) | 27.6  (23.2 to 32.6) | 0.0  (-0.1 to 0.0) | 165.6 (114.8 to 251.4) | 1.1 (0.8 to 1.6) | -0.4 (-0.7 to 0.1) | 5881.3 (3960.6 to 9218.3) | 23.6 (17.2 to 33.7) | -0.4 (-0.7 to 0.1) |
| Senegal | 2756.7  (2323.9 to 3315.5) | 25.5  (21.9 to 29.8) | 0.0  (0.0 to 0.0) | 276.5 (176.3 to 426.0) | 3.2 (2.1 to 5.0) | 0.0 (-0.4 to 0.4) | 10293.6 (6363.3 to 16079.8) | 104.5 (65.9 to 160.9) | 0.0 (-0.4 to 0.5) |
| Serbia | 4387.9  (3872.2 to 4968.6) | 35.5  (31.3 to 40.3) | -0.1  (-0.2 to -0.1) | 398.9 (258.1 to 520.3) | 2.7 (1.8 to 3.5) | -0.1 (-0.4 to 0.3) | 9545.1 (6647.3 to 12428.6) | 71.1 (50.6 to 92.1) | -0.2 (-0.4 to 0.1) |
| Seychelles | 27.4  (22.3 to 32.9) | 23.8  (19.8 to 28.4) | 0.0  (0.0 to 0.0) | 1.4 (0.8 to 2.5) | 1.3 (0.7 to 2.2) | -0.1 (-0.3 to 0.2) | 50.3 (29.3 to 88.1) | 42.4 (25.3 to 73.4) | -0.1 (-0.4 to 0.1) |
| Sierra Leone | 1458.8  (1210.5 to 1755.1) | 25.4  (21.6 to 29.6) | 0.1  (0.1 to 0.1) | 150.1 (98.1 to 229.4) | 3.4 (2.3 to 5.2) | 0.3 (-0.3 to 1.3) | 5860.3 (3778.7 to 9010.6) | 113.1 (73.3 to 172.0) | 0.3 (-0.3 to 1.4) |
| Singapore | 1303.9  (1248.0 to 1362.1) | 17.2  (16.4 to 18.0) | 0.4  (0.4 to 0.4) | 27.7 (22.8 to 31.8) | 0.4 (0.3 to 0.4) | -0.5 (-0.6 to -0.4) | 767.9 (635.0 to 925.1) | 10.1 (8.4 to 12.2) | -0.5 (-0.6 to -0.4) |
| Slovakia | 4950.1  (4475.9 to 5467.4) | 68.4  (61.9 to 75.2) | -0.1  (-0.2 to -0.1) | 237.4 (176.7 to 312.7) | 2.8 (2.1 to 3.7) | -0.3 (-0.5 to 0.0) | 6778.3 (5026.0 to 8995.5) | 86.9 (64.4 to 116.2) | -0.3 (-0.6 to -0.1) |
| Slovenia | 1236.8  (1119.4 to 1361.1) | 38.8  (35.3 to 42.7) | -0.2  (-0.2 to -0.2) | 55.6 (42.5 to 73.1) | 1.3 (1.0 to 1.7) | -0.5 (-0.7 to -0.3) | 1209.8 (935.0 to 1574.4) | 34.5 (26.5 to 45.5) | -0.6 (-0.7 to -0.4) |
| Solomon Islands | 109.5  (89.1 to 134.4) | 24.0  (19.9 to 28.5) | 0.0  (-0.1 to 0.0) | 4.8 (3.1 to 7.5) | 1.2 (0.8 to 1.7) | -0.1 (-0.4 to 0.3) | 209.8 (125.4 to 342.8) | 42.1 (27.3 to 64.1) | -0.1 (-0.4 to 0.3) |
| Somalia | 2412.6  (1929.8 to 2981.5) | 19.6  (16.3 to 23.3) | 0.0  (0.0 to 0.0) | 97.7 (55.3 to 181.8) | 1.2 (0.7 to 2.4) | -0.2 (-0.4 to 0.2) | 3924.6 (2273.7 to 7048.3) | 36.7 (20.9 to 68.2) | -0.1 (-0.4 to 0.2) |
| South Africa | 11714.6  (9672.5 to 14190.0) | 22.2  (18.5 to 26.6) | 0.0  (0.0 to 0.0) | 388.6 (314.2 to 468.8) | 0.8 (0.7 to 1.0) | -0.2 (-0.4 to 0.0) | 14257.9 (11599.2 to 17486.4) | 26.2 (21.2 to 31.8) | -0.2 (-0.4 to 0.0) |
| South Sudan | 1211.3  (980.8 to 1495.9) | 19.8  (16.4 to 23.8) | 0.0  (0.0 to 0.0) | 47.5 (24.1 to 91.3) | 1.1 (0.6 to 2.2) | -0.2 (-0.4 to 0.2) | 1696.8 (898.4 to 3128.8) | 31.8 (16.7 to 59.7) | -0.2 (-0.5 to 0.2) |
| Spain | 17925.0  (15419.2 to 20572.8) | 25.3  (21.5 to 29.3) | -0.2  (-0.3 to -0.2) | 1592.0 (1358.6 to 1819.9) | 1.5 (1.3 to 1.7) | -0.3 (-0.4 to -0.2) | 27236.6 (24093.0 to 31431.5) | 31.8 (28.2 to 37.7) | -0.4 (-0.5 to -0.3) |
| Sri Lanka | 4694.1  (3835.0 to 5655.9) | 19.4  (15.9 to 23.3) | -0.1  (-0.1 to -0.1) | 97.1 (66.7 to 135.8) | 0.4 (0.3 to 0.6) | -0.5 (-0.7 to -0.2) | 3163.3 (2213.7 to 4408.1) | 12.9 (9.1 to 17.8) | -0.5 (-0.7 to -0.3) |
| Sudan | 7298.5  (5988.8 to 8892.4) | 26.5  (22.2 to 31.2) | 0.0  (0.0 to 0.0) | 166.2 (82.9 to 262.7) | 1.0 (0.4 to 1.6) | 0.0 (-0.3 to 0.6) | 4933.1 (2829.8 to 7714.0) | 21.4 (11.6 to 33.5) | 0.0 (-0.4 to 0.5) |
| Suriname | 177.1  (147.4 to 210.3) | 29.6  (24.7 to 35.2) | 0.0  (0.0 to 0.0) | 9.9 (7.4 to 12.7) | 1.6 (1.2 to 2.1) | 0.0 (-0.3 to 0.3) | 326.8 (242.2 to 426.4) | 53.2 (39.7 to 69.2) | -0.1 (-0.3 to 0.3) |
| Sweden | 6186.6  (5182.8 to 7286.1) | 43.7  (36.6 to 51.7) | 0.2  (0.1 to 0.2) | 184.0 (137.8 to 207.6) | 0.9 (0.6 to 1.0) | -0.1 (-0.2 to 0.0) | 3804.4 (2970.6 to 4270.7) | 22.8 (17.8 to 25.8) | -0.2 (-0.3 to -0.1) |
| Switzerland | 3200.6  (2895.4 to 3539.8) | 25.0  (22.6 to 27.6) | 0.0  (0.0 to 0.0) | 105.8 (89.1 to 128.5) | 0.6 (0.5 to 0.7) | -0.2 (-0.3 to 0.0) | 2145.7 (1832.2 to 2458.0) | 14.3 (12.2 to 16.4) | -0.2 (-0.4 to -0.1) |
| Syrian Arab Republic | 3542.5  (2904.5 to 4270.6) | 26.3  (22.0 to 31.2) | 0.0  (-0.1 to 0.0) | 69.5 (32.2 to 105.4) | 0.7 (0.3 to 1.0) | -0.3 (-0.6 to 0.1) | 1887.9 (964.3 to 2859.9) | 15.3 (7.8 to 22.8) | -0.3 (-0.6 to 0.0) |
| Taiwan (Province of China) | 20027.3  (17862.7 to 22441.8) | 58.6  (52.6 to 65.4) | 0.1  (0.1 to 0.1) | 343.0 (262.8 to 439.9) | 0.9 (0.7 to 1.2) | -0.2 (-0.4 to 0.1) | 10116.7 (7844.8 to 12876.7) | 30.3 (23.4 to 38.6) | -0.1 (-0.3 to 0.2) |
| Tajikistan | 2148.1  (1736.5 to 2589.7) | 29.3  (24.1 to 34.6) | 0.0  (0.0 to 0.0) | 35.0 (25.1 to 46.2) | 0.9 (0.5 to 1.3) | 0.3 (-0.2 to 0.9) | 1217.4 (927.6 to 1760.9) | 20.3 (14.8 to 26.3) | 0.0 (-0.3 to 0.4) |
| Thailand | 20654.3  (16880.4 to 24536.1) | 22.9  (18.9 to 27.4) | 0.0  (0.0 to 0.0) | 1254.5 (857.8 to 1719.8) | 1.3 (0.9 to 1.8) | -0.2 (-0.5 to 0.2) | 40150.4 (27883.2 to 56150.4) | 44.9 (31.4 to 62.5) | -0.2 (-0.5 to 0.3) |
| Timor-Leste | 248.5  (204.3 to 300.9) | 24.7  (20.4 to 29.4) | 0.0  (0.0 to 0.0) | 10.7 (6.2 to 17.4) | 1.3 (0.8 to 2.1) | -0.1 (-0.4 to 0.3) | 358.6 (196.7 to 609.4) | 38.3 (21.2 to 64.0) | -0.2 (-0.5 to 0.2) |
| Togo | 1451.2  (1213.7 to 1742.5) | 25.2  (21.6 to 29.3) | 0.0  (0.0 to 0.0) | 171.0 (104.0 to 265.2) | 3.6 (2.4 to 5.5) | 0.2 (-0.4 to 0.9) | 6649.1 (3904.8 to 10396.5) | 121.5 (74.0 to 187.7) | 0.3 (-0.4 to 1.0) |
| Tokelau | 0.3  (0.3 to 0.4) | 26.0  (21.5 to 31.0) | 0.0  (0.0 to 0.0) | 0.0 (0.0 to 0.0) | 1.2 (0.8 to 1.6) | -0.2 (-0.5 to 0.2) | 0.4 (0.3 to 0.6) | 34.1 (23.9 to 48.8) | -0.2 (-0.5 to 0.3) |
| Tonga | 24.4  (20.3 to 29.0) | 28.0  (23.4 to 33.2) | 0.0  (0.0 to 0.0) | 1.3 (0.9 to 1.9) | 1.6 (1.2 to 2.4) | -0.1 (-0.4 to 0.3) | 39.2 (27.4 to 60.5) | 45.3 (31.9 to 69.8) | -0.1 (-0.4 to 0.4) |
| Trinidad and Tobago | 447.5  (370.3 to 531.0) | 27.3  (22.6 to 32.7) | 0.0  (0.0 to 0.0) | 16.7 (12.0 to 22.7) | 1.0 (0.7 to 1.3) | -0.1 (-0.4 to 0.3) | 558.6 (399.4 to 759.3) | 33.0 (23.7 to 44.9) | -0.1 (-0.3 to 0.3) |
| Tunisia | 3448.9  (2820.1 to 4120.3) | 27.3  (22.7 to 32.6) | 0.0  (0.0 to 0.0) | 76.6 (47.6 to 108.2) | 0.7 (0.4 to 1.0) | -0.1 (-0.5 to 0.3) | 1815.8 (1240.4 to 2520.5) | 14.7 (10.0 to 20.3) | -0.1 (-0.5 to 0.3) |
| Turkey | 23398.9  (20422.6 to 26551.4) | 26.0  (22.8 to 29.6) | 0.0  (-0.1 to 0.0) | 832.4 (588.1 to 1047.8) | 1.0 (0.7 to 1.3) | -0.3 (-0.5 to 0.0) | 17970.9 (13569.2 to 22589.0) | 20.5 (15.4 to 25.8) | -0.4 (-0.6 to -0.1) |
| Turkmenistan | 1475.2  (1201.4 to 1764.8) | 31.4  (25.9 to 37.2) | 0.0  (0.0 to 0.0) | 62.6 (48.6 to 79.8) | 1.4 (1.1 to 1.8) | 0.5 (0.0 to 1.0) | 2425.2 (1894.1 to 3124.3) | 49.7 (39.1 to 63.8) | 0.5 (0.0 to 1.1) |
| Tuvalu | 2.8  (2.3 to 3.4) | 26.0  (21.6 to 30.8) | 0.0  (0.0 to 0.0) | 0.2 (0.1 to 0.2) | 1.5 (1.0 to 2.2) | -0.2 (-0.4 to 0.2) | 5.3 (3.5 to 7.7) | 47.6 (31.8 to 69.0) | -0.2 (-0.4 to 0.3) |
| Uganda | 5035.4  (4076.2 to 6179.7) | 20.1  (16.8 to 23.8) | 0.0  (0.0 to 0.0) | 245.4 (134.4 to 418.2) | 1.5 (0.8 to 2.5) | -0.1 (-0.4 to 0.3) | 9396.9 (5109.9 to 15817.2) | 43.6 (24.0 to 74.7) | 0.0 (-0.4 to 0.4) |
| Ukraine | 46530.7  (39204.6 to 54465.3) | 77.0  (65.5 to 90.0) | 0.1  (0.1 to 0.1) | 2819.1 (2259.6 to 3444.9) | 4.7 (3.7 to 5.7) | 0.6 (0.2 to 1.0) | 110269.4 (86891.1 to 135806.1) | 196.2 (154.3 to 243.8) | 0.7 (0.3 to 1.2) |
| United Arab Emirates | 2635.8  (1968.5 to 3389.2) | 27.5  (22.9 to 32.6) | 0.0  (0.0 to 0.0) | 40.2 (19.4 to 71.4) | 0.9 (0.4 to 1.4) | -0.3 (-0.6 to 0.1) | 1768.3 (933.5 to 3045.2) | 21.2 (10.6 to 35.3) | -0.2 (-0.6 to 0.2) |
| United Kingdom | 31122.2  (27587.8 to 35056.6) | 34.6  (30.5 to 39.0) | 0.2  (0.2 to 0.2) | 1523.9 (1385.4 to 1801.8) | 1.2 (1.1 to 1.4) | 0.0 (-0.1 to 0.2) | 34238.3 (31623.6 to 38078.9) | 33.9 (31.4 to 37.4) | 0.0 (-0.1 to 0.1) |
| United Republic of Tanzania | 7699.0  (6253.0 to 9450.1) | 20.1  (16.6 to 23.9) | 0.0  (0.0 to 0.0) | 382.5 (215.9 to 752.0) | 1.4 (0.7 to 2.7) | 0.0 (-0.4 to 0.5) | 14159.5 (8214.7 to 26821.6) | 40.7 (23.1 to 78.4) | 0.0 (-0.4 to 0.6) |
| USA | 228699.2  (210599.8 to 250345.9) | 51.5  (47.4 to 56.2) | -0.2  (-0.2 to -0.2) | 4896.9 (4495.3 to 5385.2) | 0.9 (0.9 to 1.0) | 0.0 (-0.1 to 0.0) | 133259.8 (123425.3 to 147833.0) | 29.4 (27.3 to 32.6) | -0.1 (-0.1 to 0.0) |
| United States Virgin Islands | 36.6  (30.5 to 43.4) | 27.5  (22.7 to 33.0) | 0.0  (0.0 to 0.0) | 1.8 (1.3 to 2.2) | 1.1 (0.8 to 1.4) | -0.1 (-0.4 to 0.3) | 48.5 (35.7 to 62.9) | 32.8 (23.8 to 43.6) | -0.1 (-0.4 to 0.3) |
| Uruguay | 1280.8  (1074.8 to 1502.5) | 29.6  (24.8 to 34.8) | -0.1  (-0.1 to -0.1) | 65.8 (58.4 to 78.5) | 1.3 (1.2 to 1.5) | -0.2 (-0.3 to 0.0) | 1718.6 (1529.8 to 1967.1) | 38.7 (34.4 to 44.1) | -0.2 (-0.3 to -0.1) |
| Uzbekistan | 8818.4  (7167.7 to 10662.2) | 30.4  (25.1 to 36.0) | 0.0  (0.0 to 0.0) | 345.1 (276.9 to 430.1) | 1.4 (1.1 to 1.7) | 0.4 (0.0 to 1.0) | 13879.2 (11202.1 to 17150.4) | 46.4 (37.9 to 57.1) | 0.3 (0.0 to 0.7) |
| Vanuatu | 64.9  (53.9 to 78.0) | 29.3  (24.6 to 34.4) | 0.0  (0.0 to 0.0) | 3.4 (2.3 to 5.2) | 1.8 (1.2 to 2.6) | -0.1 (-0.4 to 0.4) | 133.8 (86.0 to 206.3) | 58.0 (38.5 to 87.7) | 0.0 (-0.4 to 0.5) |
| Venezuela (Bolivarian Republic of) | 10494.3  (8612.4 to 12580.2) | 35.6  (29.3 to 42.7) | 0.0  (-0.1 to 0.0) | 340.2 (247.2 to 444.9) | 1.2 (0.9 to 1.5) | -0.1 (-0.3 to 0.3) | 10909.9 (7964.6 to 14406.9) | 36.6 (26.7 to 48.3) | -0.1 (-0.3 to 0.2) |
| Viet Nam | 31786.4  (26925.4 to 37162.6) | 30.3  (26.0 to 35.0) | 0.0  (-0.1 to 0.0) | 1056.1 (678.3 to 1818.1) | 1.2 (0.8 to 2.0) | -0.4 (-0.7 to 0.0) | 33331.8 (20712.7 to 59048.5) | 32.5 (20.7 to 56.6) | -0.4 (-0.7 to 0.0) |
| Yemen | 5560.4  (4548.2 to 6833.2) | 27.2  (22.8 to 32.3) | 0.0  (0.0 to 0.0) | 136.6 (79.3 to 216.9) | 1.1 (0.6 to 1.9) | -0.1 (-0.4 to 0.4) | 4204.3 (2681.0 to 6483.7) | 25.6 (15.6 to 40.2) | -0.1 (-0.4 to 0.3) |
| Zambia | 2359.3  (1894.2 to 2912.8) | 19.8  (16.5 to 23.5) | -0.1  (-0.1 to -0.1) | 118.4 (78.1 to 181.9) | 1.5 (1.0 to 2.2) | -0.2 (-0.5 to 0.1) | 4678.9 (3111.8 to 7093.2) | 43.7 (29.1 to 66.4) | -0.2 (-0.4 to 0.2) |
| Zimbabwe | 2145.1  (1746.3 to 2645.3) | 20.0  (16.7 to 23.9) | 0.0  (0.0 to 0.0) | 110.6 (64.7 to 162.9) | 1.4 (0.8 to 2.0) | 0.3 (-0.3 to 0.9) | 4173.4 (2441.2 to 6282.0) | 42.4 (25.1 to 62.2) | 0.4 (-0.3 to 1.2) |

EAPC: estimated annual percentage change; ASIR: age-standardized incidence rate; ASMR: age-standardized mortality rate; DALY: disability-adjusted life-year; UI: uncertainty interval.


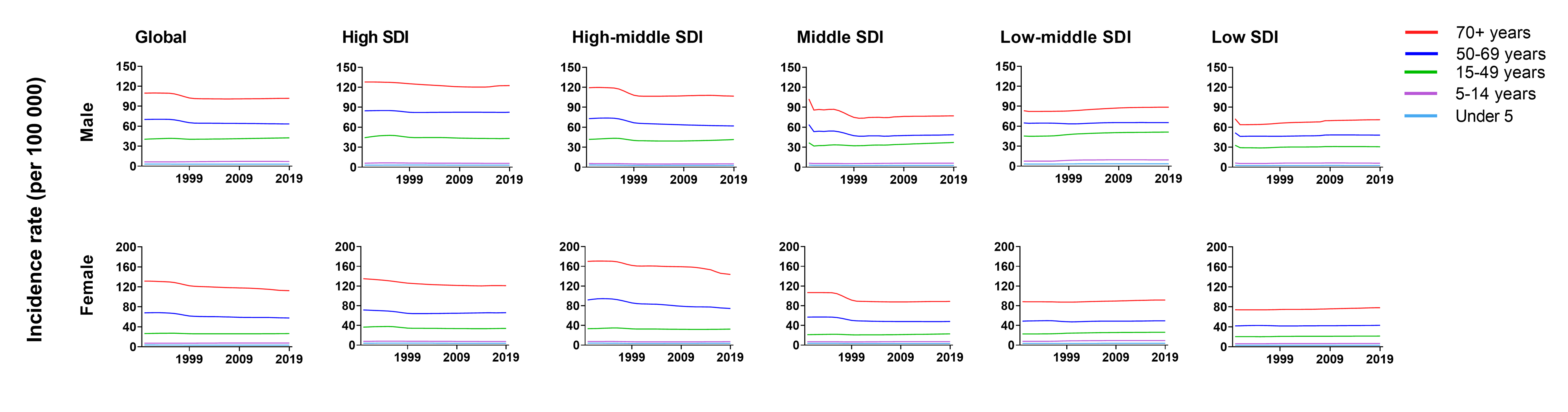


**Fig. S1.** The incidence rate (per 100 000) of acute pancreatitis in different age groups from 1990 to 2019. DALY: disability-adjusted life-year; SDI: socio demographic index.


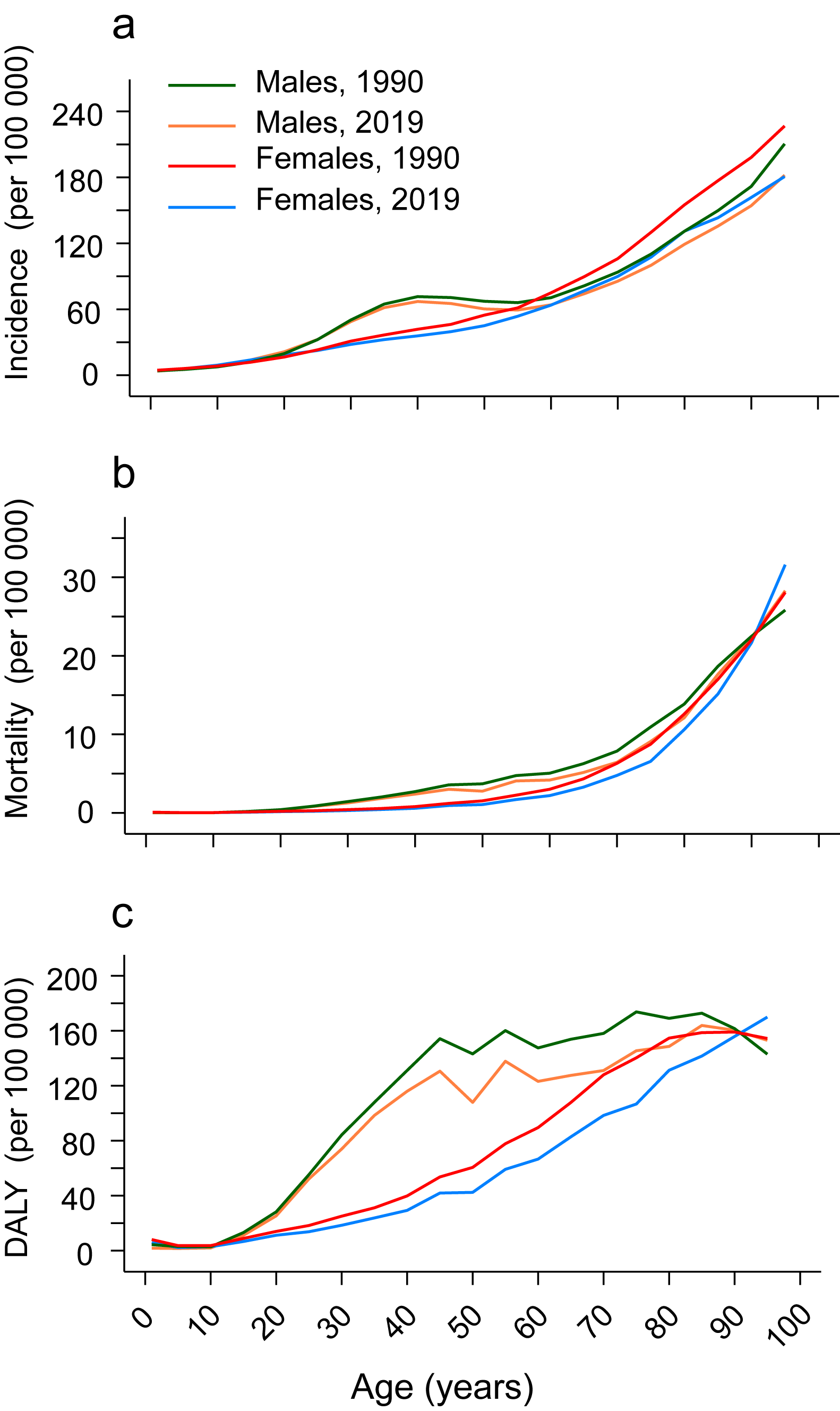
**Fig. S2** Global incidence (a), mortality (b) and DALY (c) rates per 100 000 of acute pancreatitis by age in males (men and boys) and females (women and girls) in 1990 and 2019. DALY: disability-adjusted life-year.


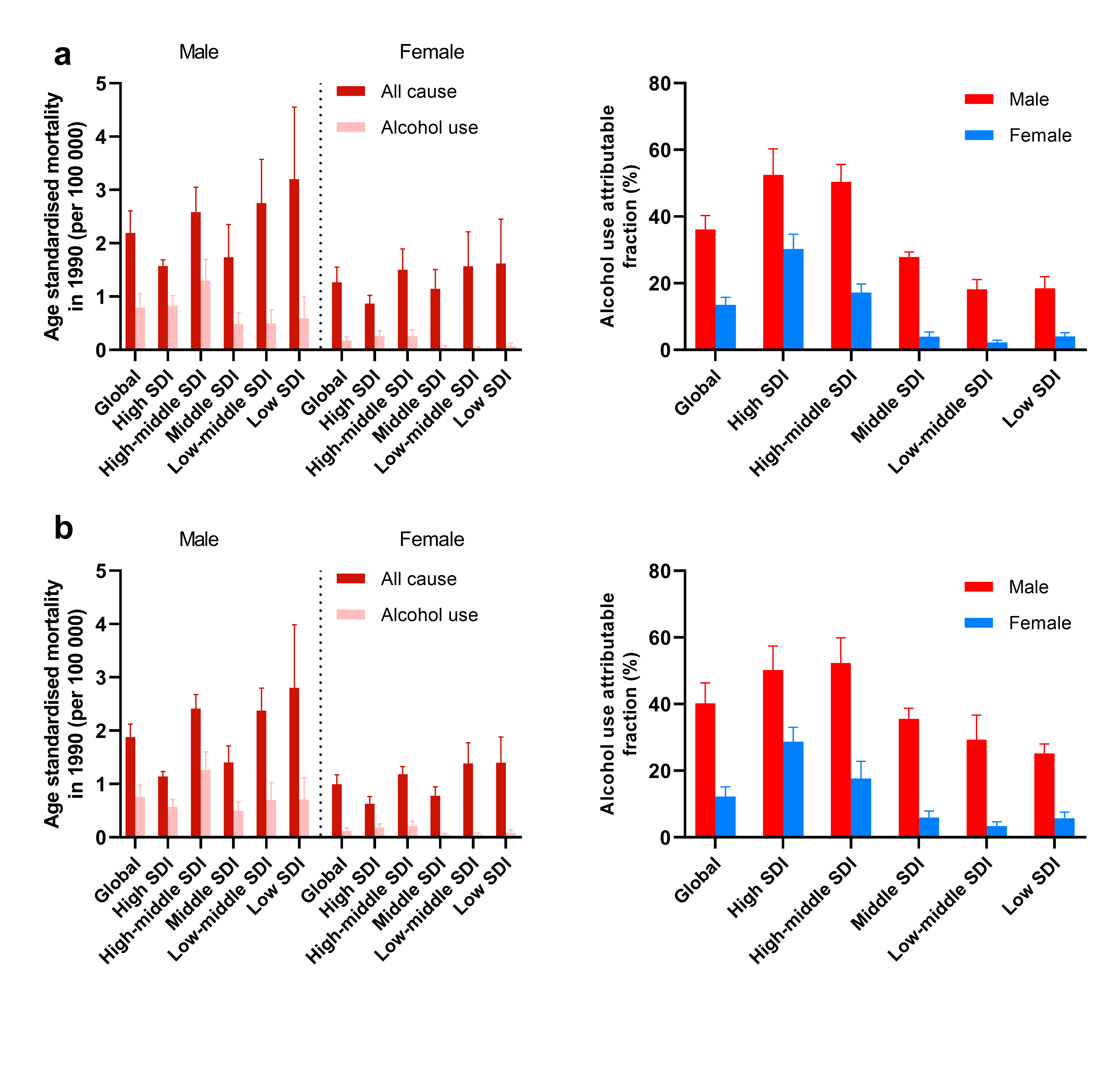
**Fig. S3** Regional distribution of age-standardized mortality rate attributed to alcohol-related acute pancreatitis in 1990 (a) and 2019 (b).


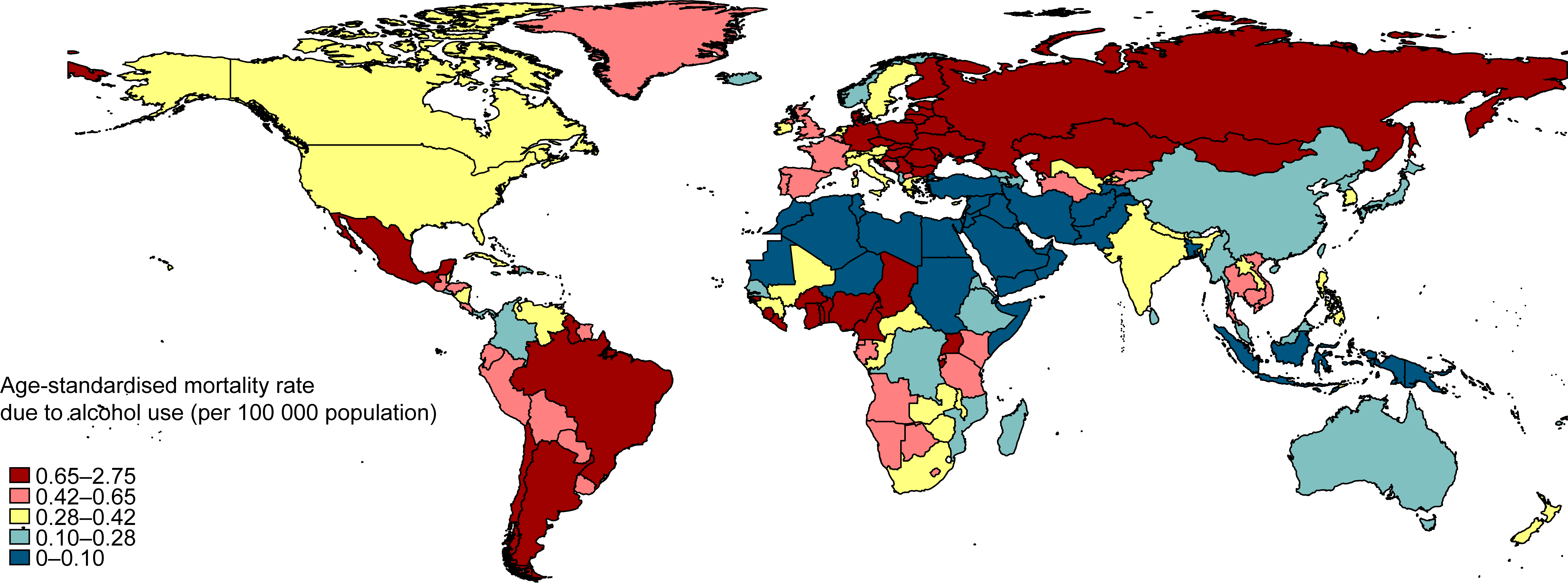
**Fig. S4** Age-standardized mortality rate of acute pancreatitis due to alcohol etiology by location, 2019.
